# Characterisation of Bacteriophage-Encoded Depolymerases Selective for Key *Klebsiella pneumoniae* Capsular Exopolysaccharides

**DOI:** 10.3389/fcimb.2021.686090

**Published:** 2021-06-18

**Authors:** George Blundell-Hunter, Mark C. Enright, David Negus, Matthew J. Dorman, Gemma E. Beecham, Derek J. Pickard, Phitchayapak Wintachai, Supayang P. Voravuthikunchai, Nicholas R. Thomson, Peter W. Taylor

**Affiliations:** ^1^ School of Pharmacy, University College London, London, United Kingdom; ^2^ Department of Life Sciences, Manchester Metropolitan University, Manchester, United Kingdom; ^3^ School of Science & Technology, Nottingham Trent University, Nottingham, United Kingdom; ^4^ Parasites and Microbes Programme, Wellcome Sanger Institute, Hinxton, United Kingdom; ^5^ Department of Medicine, University of Cambridge, Addenbrooke’s Hospital, Cambridge, United Kingdom; ^6^ Faculty of Science, Prince of Songkla University, Songkhla, Thailand; ^7^ Department of Infectious and Tropical Diseases, London School of Hygiene & Tropical Medicine, London, United Kingdom

**Keywords:** *Klebsiella pneumoniae*, bacteriophage, capsule depolymerase, whole-genome sequencing (WGS), jumbo phage, capsular polysaccharide, alternative antibacterial therapy

## Abstract

Capsular polysaccharides enable clinically important clones of *Klebsiella pneumoniae* to cause severe systemic infections in susceptible hosts. Phage-encoded capsule depolymerases have the potential to provide an alternative treatment paradigm in patients when multiple drug resistance has eroded the efficacy of conventional antibiotic chemotherapy. An investigation of 164 K*. pneumoniae* from intensive care patients in Thailand revealed a large number of distinct K types in low abundance but four (K2, K51, K1, K10) with a frequency of at least 5%. To identify depolymerases with the capacity to degrade capsules associated with these common K-types, 62 lytic phage were isolated from Thai hospital sewage water using K1, K2 and K51 isolates as hosts; phage plaques, without exception, displayed halos indicative of the presence of capsule-degrading enzymes. Phage genomes ranged in size from 41–348 kb with between 50 and 535 predicted coding sequences (CDSs). Using a custom phage protein database we were successful in applying annotation to 30 - 70% (mean = 58%) of these CDSs. The largest genomes, of so-called jumbo phage, carried multiple tRNAs as well as CRISPR repeat and spacer sequences. One of the smaller phage genomes was found to contain a putative Cas type 1E gene, indicating a history of host DNA acquisition in these obligate lytic phage. Whole-genome sequencing (WGS) indicated that some phage displayed an extended host range due to the presence of multiple depolymerase genes; in total, 42 candidate depolymerase genes were identified with up to eight in a single genome. Seven distinct virions were selected for further investigation on the basis of host range, phage morphology and WGS. Candidate genes for K1, K2 and K51 depolymerases were expressed and purified as his_6_-tagged soluble protein and enzymatic activity demonstrated against *K. pneumoniae* capsular polysaccharides by gel electrophoresis and Anton-Paar rolling ball viscometry. Depolymerases completely removed the capsule in K-type-specific fashion from *K. pneumoniae* cells. We conclude that broad-host range phage carry multiple enzymes, each with the capacity to degrade a single K-type, and any future use of these enzymes as therapeutic agents will require enzyme cocktails for utility against a range of *K. pneumoniae* infections.

## Introduction

Extracellular polysaccharide and polypeptide capsules are major virulence determinants of Gram-positive and Gram-negative pathogens, aiding colonisation of mucosal surfaces and protecting invasive bacteria from immune recognition and killing by cellular and humoral immune mechanisms during infection of susceptible hosts. They are key contributors to the enormous diversity of bacterial surfaces and provide the interface >with their immediate external environment (Roberts, 1996; Mostowy and Holt, 2018). The rising incidence of antibiotic resistance in both nosocomial and community-acquired pathogenic bacteria poses a serious threat to global health, compounded by the paucity of new antibiotics in the drug development pipeline, and has sparked renewed interest in alternative, non-antibiotic modes of treatment for bacterial infections, including the therapeutic use of bacteriophage (Lin D. M. et al., 2017), stimulation of immune cellular functions (Haney and Hancock, 2013) and the development of agents that advantageously modify the antibiotic resistance and virulence of bacterial pathogens (Mellbye and Schuster, 2011; Taylor, 2017).

There is growing evidence that rapid removal of the protective capsule during infection facilitates elimination of the invading pathogen from the host. This approach, which circumvents the consequences of acquisition of genes conferring antibiotic resistance, was initially investigated in the pre-antibiotic era by Dubos and Avery using an enzyme preparation from cultures of a peat soil bacterium to selectively remove the polysaccharide capsule from the surface of type III pneumococci (Avery and Dubos, 1930; Avery and Dubos, 1931; Dubos and Avery, 1931; Goodner et al., 1932; Francis et al., 1934). The enzyme selectively degraded the capsule; administration to mice prior to challenge with type III pneumococci gave rise to type III-specific protection; the enzyme terminated normally fatal type III pneumococcal dermal infection in rabbits and abrogated spread of the pneumonic lesion in infected cynomolgus monkeys. More recently, a depolymerase from an environmental bacterium was shown to prevent lethal infection in mice infected with a highly virulent strain of *Bacillus anthracis* (Negus et al., 2015).

Bacteriophage, predominantly of the *Podovirdae*, *Siphoviridae* and *Myoviridae* families, are a primary source of depolymerase. These enzymes usually manifest as structural proteins such as tail fibres and baseplates but may also be present as soluble proteins during the lytic cycle; they facilitate initial binding to the host bacterium and effect degradation of the capsule to initiate phage infection (Pires et al., 2016; Knecht et al., 2020). These enzymes show promise as putative therapeutic agents. Endosialidase E derived from an *Escherichia coli* K1-specific phage rapidly and selectively degraded the polysialic acid K1 capsule that enables these bacteria to cause potentially lethal sepsis and meningitis in the neonate (Tomlinson and Taylor, 1985); intraperitoneal administration of the enzyme interrupted the transit of *E. coli* K1 from gut to brain *via* the blood circulation in colonised neonatal rat pups, abrogated meningeal inflammation and prevented death from systemic infection (Mushtaq et al., 2004; Zelmer et al., 2010; Birchenough et al., 2017). Other investigations have also shown that phage-derived depolymerases have wide utility. For example, phage-encoded capsule depolymerase sensitised extensively drug-resistant *Acinetobacter baumannii* to complement and rescued normal and immunocompromised mice from lethal peritoneal sepsis (Liu et al., 2019). Depolymerase increased survival of mice infected with a lethal bolus of *Pasteurella multocida* (Chen et al., 2018) and alginate-selective enzymes can disrupt a number of bacterial biofilms (Sutherland et al., 2004).

Extended-spectrum β-lactamase-producing clones of *K. pneumoniae* have emerged as a global threat to the health of hospitalised patients and, increasingly, to otherwise healthy individuals in the community (Paczosa and Mecsas, 2016; Wyres et al., 2020). The therapeutic challenge has been compounded by the recent emergence of hypervirulent *K. pneumoniae* clones associated with pyogenic liver abscesses, pneumonia, and meningitis in young, healthy patients (Paczosa and Mecsas, 2016). In consequence, a number of reports describe the characterisation of *K. pneumoniae*-selective capsule depolymerases (recently reviewed by Knecht et al., 2020) and they demonstrate efficacy in *Galleria* (Majkowska-Skrobek et al., 2016) and murine models of infection (Lin et al., 2014, Lin H. et al., 2017; Wang et al., 2019). We have characterised 164 recent clinical isolates of *K. pneumoniae* from three hospitals in Thailand by whole-genome sequencing and phenotypic assays for virulence markers and defined the frequency of capsular (K) chemotypes (Loraine et al., 2018) with a view to developing a palette of depolymerases that would address the most abundant pathogens in intensive care settings in Thailand. A large number of distinct K types were found in low abundance although four (K2, K51, K1 and K10) were found with a frequency of >5%. Here, we report on the isolation and molecular characterisation of phage specific for K1, K2 and K51 *K. pneumoniae* and describe the properties of their depolymerases that selectively disrupt capsules associated with these serotypes.

## Materials and Methods

### Reagents

All reagents were supplied by Sigma-Aldrich, Gillingham, UK unless otherwise specified.

### Bacteria

*K. pneumoniae* clinical isolates used in this study are detailed in **Table 1**. The majority of isolates were from Thammasat University Hospital (Pathum Thani Province), Siriraj Hospital (Bangkok) and Songklanagarind Hospital (Hat Yai, Songkhla Province); these are major tertiary care hospitals in Thailand. All Thai isolates were obtained in 2016 (Loraine et al., 2018). *K. pneumoniae* isolate NTUH-K2044 has been described by Fang et al. (2004) and ATCC-43816 by Broberg et al. (2014). Bacteria were cultured in tryptic soy broth (TSB) and stored at -80°C.

**Table 1:** *K. pneumoniae* isolates used in this study.

Isolate	Location/source	Sequence type	O type	K type
SR7	Siriraj Hospital	ST23	O1v2	K1
SR65	Siriraj Hospital	ST23	O1v2	K1
TU37	Tammasat University Hospital	ST23	O1v2	K1
NTUH-K2044	National Taiwan University Hospital	ST23	O1v?	K1
SR3	Siriraj Hospital	ST14	O1v1	K2
TU18	Tammasat University Hospital	ST14	O1v1	K2
TU30	Tammasat University Hospital	ST14	O1v1	K2
SG44	Songklanagarind Hospital	ST14	O1v1	K2
SG46	Songklanagarind Hospital	ST65	O1v2	K2
SR10	Siriraj Hospital	ST65	O1v2	K2
SR51	Siriraj Hospital	ST86	O1v1	K2
ATCC-43816	American Type Culture Collection	ST493	?	K2
SR57	Siriraj Hospital	ST16	O3s	K51
TU16	Tammasat University Hospital	ST16	O3s	K51
SG43	Songklanagarind Hospital	ST16	O3s	K51
SG45	Songklanagarind Hospital	ST16	O3s	K51
SG79	Songklanagarind Hospital	ST16	O3s	K51
SR54	Siriraj Hospital	ST231	O1v2	K51
TU9	Tammasat University Hospital	ST231	O1v2	K51
TU29	Tammasat University Hospital	ST45	O3s	K10
SR4	Siriraj Hospital	ST147	O3l	K10
SG95	Songklanagarind Hospital	ST629	O3l*	K10
TU1	Tammasat University Hospital	ST36	O2v2	K102
SG41	Songklanagarind Hospital	ST307	O2v2	K102
SG56	Songklanagarind Hospital	ST307	O2v2	K102

### Phage Isolation, Amplification and Host Range

Sewage water samples were collected from the Thai hospitals: 10 ml aliquots of sewage water sample were mixed with 0.3 g TSB powder, inoculated with 100 μl overnight bacteria culture and incubated overnight at 37°C in an orbital incubator (200 rpm). After centrifugation (4,000 rpm; 20 min) supernatants were filtered (0.45 μm Merck Millipore filter) and maintained at 4°C prior to use. Individual plaques were obtained using tenfold dilutions (10^-1^ to 10^-8^) of supernatants with the double-layer agar method (Kropinski et al., 2009): 100 μl overnight culture was mixed with top agar and poured over a tryptic soy agar (TSA) plate, 10 μl supernatant was dropped onto the surface and the plates incubated overnight at 37°C. Plaques were picked with a sterile tip and transferred to 300 μl sterile SM buffer (Cold Spring Harbour, 2006) and maintained at 4°C overnight. Serial dilutions were prepared in SM buffer and used to inoculate TSB containing 200 μl logarithmic phase culture, which was then mixed with top agar, poured onto TSA plate and the plate incubated overnight at 37°C. Single plaque isolation was repeated three times: plaques from the third cycle were picked from the plates, transferred to 300 μl sterile SM buffer, maintained at 4°C overnight, serially diluted in SM buffer prior to inoculation into TSB containing 200 μl logarithmic phase bacteria, dilutions mixed with top agar and mixtures poured onto TSA for overnight incubation at 37°C. For collection of phage, 10 μl of phage stock was added to 10 ml logarithmic phase bacteria in TSB and the mixture incubated overnight at 37°C in an orbital incubator at 200 rpm. The culture was centrifuged (4,000 rpm; 4°C; 30 min) and supernatants filtered (0.45 μm). Suspensions were stored at 4°C. Phage host range was determined by spot test; 100 µl logarithmic phase bacterial culture was mixed with 10 ml 0.75% TSA, the mixture poured onto 10 ml 1.5% TSA plates and 10 µl phage lysate (~10^9^ pfu/ml) spotted onto the plate prior to overnight incubation at 37°C. All host range assays were performed in triplicate.

### Visualisation of Phage

For transmission electron microscopy (TEM), formvar/carbon grids (Agar Scientific Ltd., Stansted, UK) were prepared by glow discharge (10 mA, 10 s) using a Q150R ES sputter coater (Quorum Technologies Ltd., Lewes, UK). Bacteriophage suspensions (15 µl) were pipetted onto the surface of the grids for 30 s before removal with Whatman filter paper. Samples were then stained by pipetting 15 µl 2% phosphotungstic acid onto the grids. Excess stain was removed with Whatman filter paper and grids air dried. Samples were visualised using a JOEL JEM-2100Plus transmission electron microscope at an accelerating voltage of 160 Kv.

### Genomic DNA Extraction and Sequencing

Phage DNA was extracted using protocol 3.3.3 described by Pickard (2009). Briefly, 1.8 ml phage lysate was incubated with 10 µg/ml DNase I and 50 µg/ml RNase A for 30 min, then incubated for a further 30 min with 10 µg/ml Proteinase K and 0.5% SDS, both at 37°C. Mixtures were transferred to phase-lock Eppendorf tubes, mixed with 500 µl phenol:chloroform:isoamyl alcohol (25:24:1), centrifuged (1,500 *g*; 5 min), the aqueous phase moved to fresh phase-lock tubes and the extraction repeated. Aqueous phases were then moved to fresh phase-lock tubes and mixed with 500µl chloroform:isoamyl alcohol (24:1), centrifuged (6,000 *g*; 5 min), the aqueous phase removed, mixed with 45 µl 3M sodium acetate (pH 5.2) and 500 µL isopropanol, maintained at room temperature for 20 min, centrifuged (14,000 rpm; 20 min), the pellet washed twice with 70% ethanol, left to dry and suspended in TE buffer. DNA quality was assessed by restriction digestion with HindIII-HF (New England Biolabs UK) followed by electrophoresis on 0.8% agarose gels. In all cases, clear bands indicated absence of contamination with bacterial DNA; no ethanol contamination was detected when DNA was subjected to nanodrop analysis. Phage genomic DNA (~0.5 μg) was sequenced using Illumina HiSeq X10 paired-end sequencing. Annotated assemblies were produced according to Page et al. (2016). Sequence reads were assembled *de novo* with Velvet v1.2 (Zerbino and Birney, 2008) and VelvetOptimiser v2.2.5 (Gladman and Seemann, 2008). Assemblies were also made using SPades v1.3.1. (Nurk et al., 2017) with the -meta option to identify prophage. Reads were annotated using PROKKA v1.14.6 (Seemann, 2014) using a custom Caudovirales gene database provided by Dr Andrew Millard, University of Leicester. The stand-alone scaffolder SSPACE (Boetzer et al., 2011) was used to refine contig assembly; sequence gaps were filled using GapFiller (Boetzer and Pirovano, 2012). Phylogenetic trees were prepared using Archaeopteryx (https://sites.google.com/site/cmzmasek/home/software/archaeopteryx).

### Protein Expression and Purification

Putative depolymerase gene coding sequences were cloned directly from phage genomes using PCR (conditions in **Supplementary Table 1**) by ligation into the pET26b+ expression vector (Merck KGaA, Darmstadt, Germany) between NdeI-XhoI or HindIII-XhoI depending on the presence or absence of the pelB leader sequence to aid protein solubility. All expressed proteins carried a C-terminal-his_6_ tag. Plasmid sequences are shown in **Supplementary Data Sheet 1**. Proteins were expressed in T7 express cells (ER2766, NEB) by IPTG induction at OD_600_ ~0.4. Cells were collected by centrifugation (5,000 *g*; 4°C; 15 min), the pellet suspended in 40 ml binding buffer (50 mM NaH_2_PO_4_; 300 mM NaCl; 10 mM imidazole; pH 7.4) and one protease inhibitor cocktail tablet (Roche Applied Science) together with 100 µg/ml lysozyme. After maintenance on ice for 30 min, the lysate was centrifuged (10,000 *g*; 4°C; 30 min) and the supernatant loaded onto a 5 ml Histrap column housed in an AKTAprime FPLC system (Cytiva, Amersham, UK). Proteins were eluted with a 30 ml gradient of loading buffer into elution buffer (binding buffer with 500 mM imidazole), dialysed against storage buffer (binding buffer, no imidazole; 5 ml eluate against 5 l buffer), filtered (0.22 µM) and stored at -80°C. Protein concentration was determined by Bradford assay.

### Enzyme Characterisation

*Klebsiella pneumoniae* capsules were prepared essentially as described by Domenico et al. (1989): 200 ml bacterial culture was mixed with 40 ml 1% Zwittergent 3-14 detergent in 100 mM citric acid (pH 2.0) for 30 min at 50°C. Bacteria were removed by centrifugation (16,000 *g*; 2 min), supernatants mixed with ice cold ethanol to a final ethanol concentration of 80%, and maintained at 4°C for 30 min. After centrifugation (16,000 *g*; 5 min) pellets were air dried, suspended in 40 ml H_2_O and treated with DNase and RNase (both 30 µg/ml) for 30 min at 37°C. Preparations were lyophilised after heat inactivation (65°C; 10 min).

To determine the impact of depolymerase on capsule viscosity, enzyme aliquots (final protein concentrations: GBH001_056, 25 ng/µl; GBH038_054, 22.2 ng/µl; GBH019_279, 1.6 ng/µl) were mixed with 400 μg polymer in 1 ml protein storage buffer and incubated for 1 h at 37°C; reactions were terminated by maintenance at 98°C for 5 min and, if required, samples stored at −20°C before viscometric analysis. Polymer viscosity was determined using an Anton Paar rolling ball microviscometer (Anton Paar, Graz, Austria); samples were transferred to a glass viscometry 1.6 mm diameter capillary containing a solid steel ball. Viscosity was determined as the time taken for the ball to fall 25 cm through the sample at an angle of 20° to the horizontal; each automated, timed determination was performed six times. Degradation of polysaccharide substrate was also determined by gel electrophoresis. Enzyme aliquots were mixed with 7.5 μg of substrate in 22.5 μl reaction volume, the reactions terminated as described above and after electrophoresis using 10% SDS-PAGE, gels were washed (10% acetic acid; 25% ethanol) after 5, 10 and 15 min at 50°C, stained with 0.1% Alcian blue in 10% acetic acid and 25% ethanol for 30 min at 50°C and de-stained with wash buffer at room temperature overnight. Enzyme activity was also monitored by spotting 10 μl depolymerase onto agar plates seeded with appropriate *K. pneumoniae* strains (spot tests). The capacity of recombinant depolymerases to remove capsule from the surface of *K. pneumoniae* was examined by preparation of turbid bacterial suspensions (OD_600_ ∼ 1) in 100 μl PBS, enzyme added to give a final concentration of 1 μg/ml and incubation for 90 min at 37°C. Negative controls were incubated with PBS. Bacterial suspensions were combined with nigrosin and examined by phase contrast microscopy. Area occupied by the capsule was determined using the microbeJ application of ImageJ (https://imagej.net/Welcome).

## Results

### Phage Isolation

*K. pneumoniae* isolates TU37 (capsule K1), TU18 (K2), TU30 (K2), SG44 (K2), SG46 (K2), TU9 (K51), SG43 (K51), SG45 (K51) and SG79 (K51) (**Table 1**) were used to isolate phage selective for these capsule types from three filtered sewage water samples. Sixty-two phage were isolated, encompassing a variety of distinct host range patterns (**Supplementary Table 2**); 28 of these were chosen for further study, based on divergent morphology, host range, genome size and sequence similarity, and were ordered into seven groups based on a high degree (>95%) of nucleotide sequence identity (**Table 2**). In general, phage producing plaques on a host carrying the target K-type lysed other clinical isolates of the same K type, although there were exceptions as detailed in **Supplementary Table 2** and **Table 2**. The majority did not have absolute specificity for K-type: for example, GBH013 and GBH017, isolated on and with specificity for isolates carrying the K2 capsule type, were able to lyse all four K1 indicator strains, and the six phage isolated on and active against K51 strains also lysed two of three K102 indicator strains, producing incomplete lysis of the third. Incomplete lysis was independent of virion concentration and is therefore not due to “lysis from without”. All lytic plaques on fully-susceptible host strains were surrounded by halos after overnight incubation at 37°C followed by 3-4 days storage at 4°C, indicating diffusion of soluble (non-virion) capsule depolymerase into the surrounding bacterial lawn (Adams and Park, 1956). Representative phage from each of the seven groups shown in **Table 2** were also examined against *K. pneumoniae* expressing capsular K-types K5, K20, K15-1, K21, K24, K25, K28, K54, K103 and K122. GBH029 and GBH054 lysed the K21 isolate SR95 and GBH001, GBH014, GBH029 and GBH033 lysed the K28 isolate SR33; in all other cases no lytic activity was noted.

**Table 2:** *K. pneumoniae* phage isolates sequenced in this study, showing patterns of lytic activity against various *K. pneumoniae* K-types.

Phage	Host^a^	kb^b^	SR7	SR65	TU37	NTUH-K2044	SR3	TU18	SG44	SR10	SR51	ATCC-43816	SR57	TU16	SG45	SR45	TU9	TU29	SR4	SG95	TU1	SG41	SG56
			K1				K2						K51					K10			K102		
**GBH001**	**TU37**	**45.96**	**++** ^c^	**+**	**++**	**++**	**-**	**-**	**-**	**+**	**-**	**-**	**-**	**+**	**-**	**-**	**-**	**-**	**-**	**-**	**-**	**-**	**-**
GBH002	TU37	45.97	**++**	**+**	**++**	**++**	**-**	**-**	**-**	**-**	**-**	**-**	**-**	**+**	**-**	**-**	**-**	**-**	**-**	**-**	**-**	**-**	**-**
GBH003	TU37	45.96	**++**	**+**	**++**	**+**	**-**	**-**	**-**	**-**	**-**	**-**	**-**	**+**	**-**	**-**	**+**	**-**	**-**	**-**	**-**	**-**	**-**
GBH005	TU37	45.96	**++**	**+**	**++**	**++**	**-**	**-**	**-**	**-**	**-**	**-**	**-**	**+**	**-**	**-**	**-**	**-**	**-**	**-**	**-**	**-**	**-**
GBH007	TU37	45.96	**++**	**+**	**++**	**++**	**-**	**-**	**+**	**++**	**-**	**-**	**-**	**+**	**-**	**-**	**-**	**-**	**-**	**-**	**-**	**-**	**-**
**GBH014**	**SG44**	**41.70**	**+**	**-**	**-**	**-**	**++**	**++**	**++**	**++**	**++**	**++**	**-**	**-**	**-**	**-**	**-**	**-**	**-**	**-**	**-**	**-**	**-**
GBH013	SG44	41.70	**++**	**++**	**++**	**++**	**++**	**++**	**++**	**++**	**++**	**++**	**-**	**+**	**-**	**-**	**-**	**-**	**-**	**-**	**-**	**-**	**-**
GBH017	SG44	41.09	**++**	**++**	**++**	**++**	**++**	**++**	**++**	**++**	**++**	**++**	**-**	**+**	**-**	**-**	**-**	**-**	**-**	**-**	**-**	**-**	**-**
GBH018	SG44	40.87	**-**	**-**	**-**	**-**	**++**	**++**	**++**	**++**	**-**	**++**	**-**	**++**	**-**	**-**	**-**	**-**	**-**	**-**	**-**	**+**	**-**
**GBH019**	**TU9**	**347.55**	**-**	**-**	**-**	**-**	**-**	**-**	**+**	**-**	**-**	**-**	**++**	**++**	**++**	**++**	**++**	**-**	**-**	**-**	**+**	**++**	**++**
GBH020	TU9	347.54	**-**	**-**	**-**	**-**	**-**	**-**	**-**	**-**	**-**	**-**	**++**	**++**	**++**	**++**	**++**	**-**	**-**	**-**	**+**	**++**	**++**
GBH023	TU9	347.55	**-**	**-**	**-**	**-**	**-**	**-**	**-**	**-**	**-**	**-**	**++**	**++**	**++**	**++**	**++**	**-**	**-**	**-**	**+**	**++**	**++**
**GBH029**	**SG43**	**49.92**	**-**	**-**	**-**	**-**	**-**	**-**	**++**	**+**	**-**	**+**	**-**	**++**	**-**	**-**	**-**	**-**	**+**	**-**	**-**	**-**	**-**
GBH026	SG43	50.49	**-**	**-**	**-**	**-**	**-**	**++**	**++**	**+**	**-**	**++**	**-**	**++**	**-**	**+**	**+**	**-**	**+**	**-**	**-**	**+**	**-**
GBH027	SG43	50.49	**-**	**-**	**-**	**-**	**-**	**++**	**++**	**+**	**-**	**+**	**-**	**++**	**-**	**+**	**+**	**-**	**+**	**-**	**-**	**+**	**-**
**GBH033**	**SG45**	**165.75**	**-**	**-**	**-**	**-**	**-**	**-**	**+**	**-**	**-**	**+**	**-**	**++**	**+**	**+**	**-**	**-**	**-**	**-**	**-**	**-**	**-**
GBH035	SG45	165.75	**-**	**-**	**-**	**-**	**-**	**-**	**++**	**+**	**-**	**+**	**-**	**++**	**+**	**+**	**-**	**-**	**-**	**-**	**-**	**-**	**-**
**GBH038**	**SG46**	**43.73**	**-**	**-**	**-**	**-**	**++**	**++**	**++**	**++**	**++**	**++**	**-**	**-**	**-**	**-**	**-**	**-**	**-**	**-**	**-**	**-**	**-**
GBH039	SG46	43.73	**-**	**-**	**-**	**-**	**++**	**++**	**++**	**++**	**++**	**++**	**-**	**-**	**-**	**-**	**-**	**-**	**-**	**-**	**-**	**-**	**-**
GBH045	TU18	44.03	**-**	**-**	**-**	**-**	**++**	**++**	**++**	**++**	**++**	**++**	**-**	**-**	**-**	**-**	**-**	**-**	**-**	**-**	**-**	**-**	**-**
GBH046	TU18	44.65	**-**	**-**	**-**	**-**	**++**	**++**	**++**	**++**	**++**	**++**	**-**	**-**	**-**	**-**	**-**	**-**	**-**	**-**	**-**	**-**	**-**
GBH049	TU30	44.22	**-**	**-**	**-**	**-**	**++**	**++**	**++**	**++**	**++**	**++**	**-**	**-**	**-**	**-**	**-**	**-**	**-**	**-**	**-**	**-**	**-**
GBH050	TU30	44.20	**-**	**-**	**-**	**-**	**++**	**++**	**++**	**++**	**++**	**++**	**-**	**-**	**-**	**-**	**-**	**-**	**-**	**-**	**-**	**-**	**-**
**GBH054**	**SG79**	**58.59**	**-**	**-**	**-**	**-**	**-**	**++**	**++**	**++**	**+**	**-**	**-**	**++**	**++**	**-**	**++**	**-**	**-**	**-**	**-**	**-**	**-**
GBH055	SG79	58.52	**-**	**-**	**-**	**-**	**-**	**++**	**++**	**++**	**-**	**-**	**+**	**++**	**++**	**+**	**++**	**-**	**-**	**-**	**-**	**-**	**-**
GBH056	SG79	58.52	**-**	**-**	**-**	**-**	**-**	**++**	**++**	**++**	**-**	**-**	**+**	**++**	**+**	**-**	**++**	**-**	**-**	**-**	**-**	**-**	**-**
GBH060	SG79	58.52	**-**	**-**	**-**	**-**	**-**	**-**	**++**	**++**	**-**	**-**	**+**	**++**	**++**	**-**	**++**	**-**	**+**	**-**	**-**	**-**	**-**
GBH061	SG79	58.52	**-**	**-**	**-**	**-**	**-**	**-**	**+**	**++**	**-**	**-**	**++**	**++**	**++**	**+**	**++**	**-**	**-**	**-**	**-**	**-**	**-**

a clinical isolates detailed in **Table 1**.

b genome length determined from whole-genome data.

c clear plaque, ++; +, incomplete lysis; -, no lysis. Determined by spot assay, phage concentration ~10_10_ virions/ml.

The 28 phages selected fell into seven groups based on sequence similarity of >95%. Exemplars for each group in bold.

**Figure 1:**
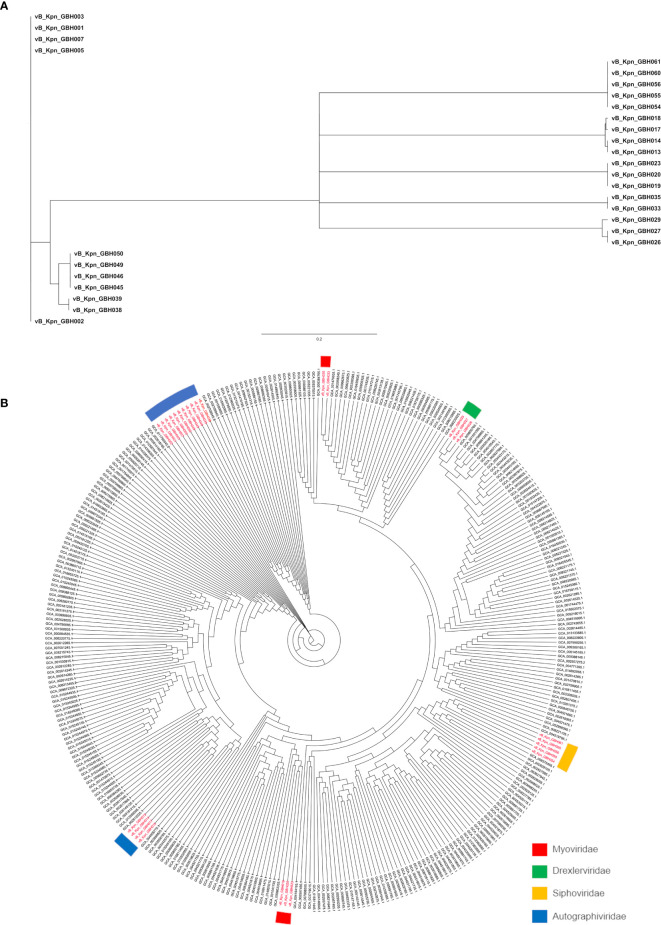
Phylogeny of Thai *K pneumoniae-* selective phage. **(A)** MASH tree with degree of genetic similarity amongst the 28 phage genomes sequenced in this study. **(B)** Phylogenetic tree prepared using Archaeopteryx showing the relationship of the 28 phage genomes (in red) to 314 published *Klebsiella* phage genomes.

**Figure 2:**
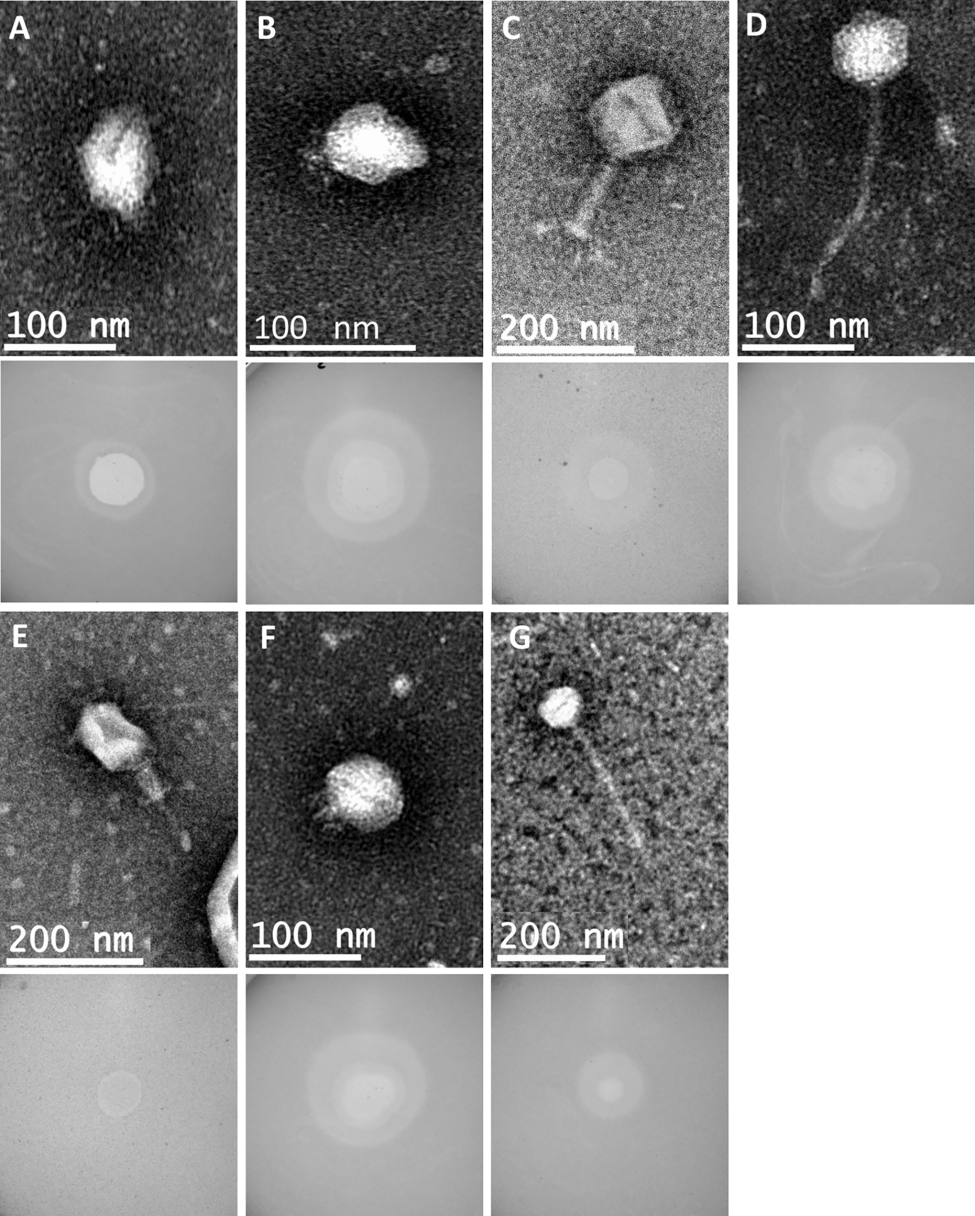
Representative transmission electron micrographs and plaque morphology of the seven exemplars for each of the genetically distinct phage groups defined in this study and shown in **Table 2**. **(A)** GBH001 (mean capsid diameter 63.4 nm; mean tail length N/A; *n* = 10) **(B)** GBH014 (64.0 nm; N/A; *n* = 7) **(C)** GBH019 (132.7 nm; 163.4 nm; *n* = 9) **(D)** GBH029 (73.8 nm; 188.8 nm; *n* = 7) **(E)** GBH033 (86.7 nm; 105.2 nm; *n* = 8) **(F)** GBH038 (72.0 nm; N/A; *n* = 6) **(G)** GBH054 (81.2 nm; 256.4 nm; *n* = 11).

The genetic relatedness of the 28 phage genomes sequenced in this study (**Figure 1A**) in comparison to the 314 from published studies of *Klebsiella* phage is shown in **Figure 1B**. **Figure 1A** uses formal designations for the phage (e.g., vB_KpnA_GBH001) but here and elsewhere in this study the abbreviated form has been used (e.g., GBH001) for clarity (Adriaenssens and Brister, 2017). The *Klebsiella* phage genome diversity found in our study paralleled that in studies of previously sequenced phage (Santiago and Donlan, 2020; Townsend et al., 2021). A combination of genome sequence comparison and TEM was employed to classify the phage in accord with the most recent International Committee on Taxonomy of Viruses (ICTV) guidelines (Adriaenssens et al., 2020); five (GBH054, GBH055, GBH056, GBH060, GBH061) were assigned to the family *Siphoviridae* (order *Caudovirales*), five (GBH019, GBH020, GBH023, GBH033, GBH035) to the *Myoviridae* (order *Caudovirales*), fifteen (GBH001, GBH002, GBH003, GBH005, GBH007, GBH013, GBH014, GBH017, GBH018, GBH038, GBH039, GBH045, GBH046, GBH049, GBH050) to the *Autographiviridae* (order *Tubulavirales*) and three (GBH026, GBH027, GBH029), to the *Drexlerviridae* (order *Tubulavirales*). There was complete concordance between assignments based on genome sequence and morphology as determined by TEM; representative images from each phage family together with corresponding plaque morphology are shown in **Figure 2**.

From the genome sequence data the 28 phage showed a wide range of genome sizes (**Table 2**). Four phage selective for either capsular K1 or K2 strains possessed genomes of <50 kb, seven genomes of 50-100 kb and seven genomes of 100-200 kb. Three K51-selective phage displayed non-identical genomes of 348 kb and consequently fell into the category of jumbo (Yuan and Gao, 2017) also known as huge (Al-Shayeb et al., 2020) phage characterised by genomes of >200 kb. As isolation-based methods select against large phage (Yuan and Gao, 2017), only 94 jumbo phage have been described following isolation and only three of these, with a high degree of sequence similarity, are *Klebsiella* spp virions (Šimoliūnas et al., 2013; Pan et al., 2017; Mora et al., 2021): these have genomes of 345.8 kb, making phage GBH019, GBH020 and GBH023 (**Table 2**) amongst the largest *Klebsiella* phage described and sequenced to date.

### Phage Genome Analysis

The 28 phage selected for sequencing were found to belong to seven groups with members sharing MASH (Ondov et al., 2016) DNA sequence similarity of >95%. These groups are exemplified by phage GBH001 (45 kb), GBH014 (41 kb), GBH019 (348 kb), GBH029 (50 kb), GBH033 (166 kb), GBH038 (44 kb) and GBH054 (59 kb). Phage GBH038 and GBH50 share 96.5% MASH sequence identity but have divergent gene orders. As others have noted (Townsend et al., 2021), analysis of genome assemblies using metaSPAdes showed the presence of prophage in sequenced lysate preparations of lytic phage GBH001 (prophage genome size 48.6 kb) and GBH029 (43.4 kb) and closely related phage. However, these were low coverage assemblies compared to those of the lytic phage with coverage depths of <7% compared to the main phage assembly. Presumably these prophage were released from the host genome during phage lysate preparation. Prophage from *K. pneumoniae* on which GBH001 formed plaques were 48.6 kb in length and contained genes associated with integration and excision from host genomes such as integrases and transposases, and a *cro* repressor protein. GBH029 and similar phage contain a prophage of 43.4 kb whose genomes contain integrase, transposase and putative excisionase genes.

### Jumbo Phage GBH019

**Figure 3:**
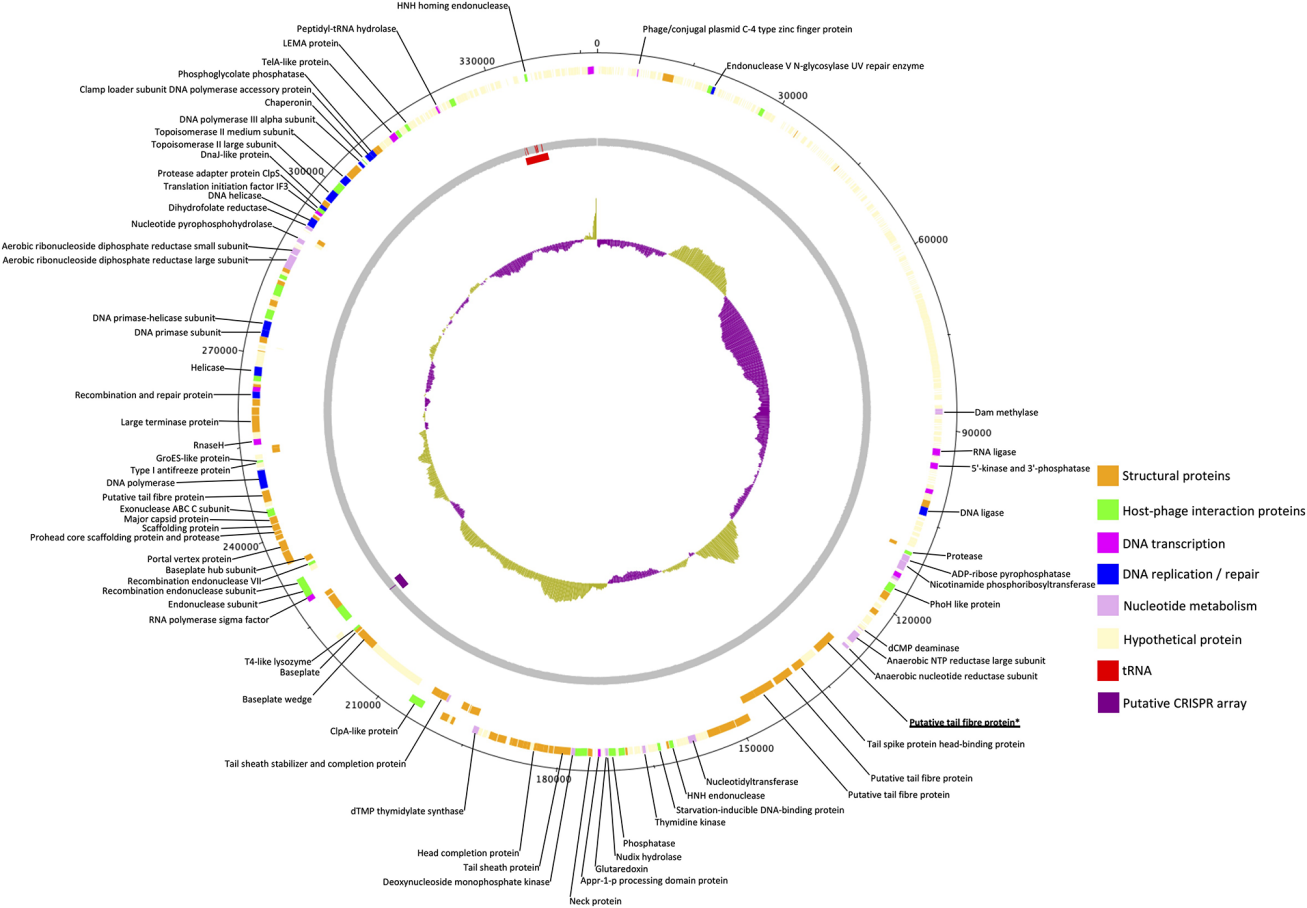
Circular genomic map of Jumbo phage GBH019. Genes involved in virion structure (orange), host interaction (green), nucleotide metabolism (pink), DNA replication and repair (blue), and DNA transcription (magenta) are shown as the positions of six tRNAs (red) and a putative CRISPR array (purple). %GC is shown in the centre of the figure. Hypothetical proteins are shown in cream. Figure generated using DNA Plotter release 18.1.0.

The genome of phage GBH019, at 347,546 bp, is the largest of any *K. pneumoniae* phage sequenced to date. It contains 534 CDSs and six tRNA genes with a coding gene density of 90.2% and GC% of 32.0. Annotations were attributed to 141 CDSs (shown in colour in **Figure 3**) leaving a total of 393 (73.6%) CDSs of unknown function. GBH019 shows high similarity to previously described jumbo phage Muenster (Mora et al., 2021), K64-1 (Pan et al., 2017) and vB_KleM-RaK2 (Šimoliūnas et al., 2013) with DNA similarity values calculated using MASH of 98.2–98.4% but with highly dissimilar gene organisation (**Supplementary Figure 1**). Examination of the pangenome of these four phage using Roary 3.13 (Page et al., 2015) showed that 384 genes were common to all genomes (BLASTP > 90%). The genome of GBH019 has three putative tail fibre protein genes (**Figure 3**) as well as tail spike and other tail-associated protein genes. These genes encode structures involved in host receptor recognition and binding, and the presence of several such structures in the genomes of these large phage is associated with extended host ranges (Pan et al., 2017). One of these putative tail fibre genes, corresponding to GBH019 gene 00383 is common to all four phage and is highly conserved (>98% DNA sequence identity, determined by Clustal Φ).

GBH019 contains six tRNA genes in common with those of phage Muenster, with K64-1 having seven and KleM-RaK2 eight. GBH019 also has many genes involved in nucleotide metabolism, DNA transcription, replication and repair including a DNA polymerase and DNA and RNA ligases and at least two endonucleases (**Figure 3**). These genes are presumably involved in sophisticated re-purposing of the host DNA and protein synthesis machinery to phage particle biosynthesis. GBH019, in common with the three other similar phage has a putative CRISPR array of the sequence CCCAGTATTCATGCGGGTTGTAGGAATTAGGGACAC although no Cas genes are present in these genomes.

### Phage GBH033

With the second largest phage genome in this study (165,752bp), GBH033 is similar to several *Myoviridae* genomes (>95% BLASTN nucleotide similarity) including *Klebsiella* virus JD18 (Genbank accession number KT239446). It has an AT-rich genome with %GC of 39.5 and contains 290 CDSs which we annotated with functions or putative functions. The genome of GBH033 contains 16 tRNA genes, the same number as found in the genome of phage JD18.

### Phage GBH001 and GBH038

Phage GBH001 and GBH038 have genome sizes of 44,964 and 43,726 bp respectively. They share 93.4% DNA similarity, determined using MASH, and have 56 and 54 CDSs respectively. We ascribed functions or putative functions to 31 of 56 GBH001 and 29 of 54 GBH038 genes. Their genomes are more GC-rich than that of GBH019 with GC% of 54.2 and 54.3, compared to 32.2. The most closely related genomes of both phage determined by BLASTN homology are phage of the family *Autographiviridae* (subfamily *Slopekvirinae*, genus *Drulisvirus*) whose genomes share <92% BLASTN DNA similarity and therefore both phage may represent a new species within this subfamily as <95% BLASTN similarity equates to new species (Adriaenssens et al., 2020).

### Phage GBH014

The genome length of phage GBH014 is 40,697 bp, with GC% of 53.2, and the phage shares 96.2% BLASTN similarity (over 85% of its genome) with the *Klebsiella Autographidae* phage 066028 (Genbank accession MW042796.1) that is currently designated as an unclassified *Przondovirus*. However, GBH014 may represent a new species of *Klebsiella* phage as it shares <5% DNA similarity over its whole genome with any phage genome described thus far. We were able to annotate functions or putative functions to 25 of the 51 predicted CDSs in the GBH014 genome.

### Phage GBH029

GBH029 has a genome of 49,924 bp, a GC% of 51.2% and is most similar to *Klebsiella* phage Sweeney (Genbank accession NC_049839.1) which is an unclassified *Webervirus* in the family *Drexlerviridae*. Functions or putative functions were annotated to 35 of the 77 CDSs in this genome.

### Phage GBH054

The genome of phage GBH054 is 58,588 bp long and has a GC% of 56.2. Functions or putative functions could only be annotated to 28 of 79 CDSs in this genome. This genome is most similar to that of the *Siphoviridae* phage Soft (Genbank accession MN106244.1). BLASTN comparison of both genomes revealed 93.84% sequence identity over 84% of the GBH054 genome, indicating that this phage may be a new species of *Siphovirus*.

### Identification and Expression of Phage Depolymerase Genes

To identify genes encoding depolymerases with the capacity to degrade capsular polysaccharides produced by *K. pneumoniae* K1, K2 and K51 isolates, BLASTX (States and Gish, 1994) was used to search a customised library (**Supplementary Table 3**) of 25 published phage-encoded *K. pneumoniae* capsule depolymerases.

Phage GBH001 displayed lytic activity against the four clinical isolates of K1 capsule type (**Table 2**). The search identified two potential GBH001-encoded depolymerase genes, GBH001_048 (541 residues) and GBH001_056 (651 residues). The former was matched to multiple tail fibre and tail spike proteins, and to other putative depolymerase proteins using JACKHMMER (Finn et al., 2015); we also identified an N-terminal match with a tail fibre protein using the EMBL protein family database InterProScan (Quevillon et al., 2005). It was cloned and expressed as described in *Materials and Methods* but displayed no capsule depolymerase activity against K1 capsule. The latter showed >96% identity with known K1-selective depolymerases, was matched to tail fibre proteins using JACKHMMER (Finn et al., 2015) with the reference proteomes database and contained a pectin-lyase fold (residues 265-568) as determined with the EMBL protein family database InterProScan. GBH001_056 was inserted between pET26b+ NdeI and XhoI sites and grown for 16 h at 26°C following induction with 0.01 mM IPTG at OD_600_ ~0.4. Spot tests indicated that the his_6_-tagged, affinity-purified protein possessed depolymerase activity against the K1 capsule but not against K2, K51, K10 and K102 capsules (**Table 3**). Depolymerase GBH001_056 possessed no activity against Thai clinical isolates expressing the K20, K24, K15-1, K21, K25, K103, K122, K28, K5, K54, K62 and K74 capsules; these K-types were found with relatively high frequency (incidence >1.5%) in clinical isolates from our Thai *K. pneumoniae* collection (Loraine et al., 2018).

**Table 3:** Spot tests showing the capacity of individual depolymerases to degrade the most common *K. pneumoniae* K-types encountered in Thai hospitals (Loraine et al., 2018).

Depolymerase	Strain/isolate; capsule (K) type																				
	SR7	SR65	TU37	NTUH-K2044	SR3	TU18	SG44	SR10	SR51	ATCC-43816	SR57	TU16	SG45	SR54	TU9	TU29	SR4	SG95	TU1	SG41	SG56
	K1				K2						K51					K10			K102		
**GBH001_056**	**+**	**+**	**+**	**+**	**-**	**-**	**-**	**-**	**-**	**-**	**-**	**-**	**-**	**-**	**-**	**-**	**-**	**-**	**-**	**-**	**-**
GBH038_054	**-**	**-**	**-**	**-**	**+**	**+**	**+**	**+**	**+**	**+**	**-**	**-**	**-**	**-**	**-**	**-**	**-**	**-**	**-**	**-**	**-**
GBH019_279	**-**	**-**	**-**	**-**	**-**	**-**	**-**	**-**	**-**	**-**	**+**	**+**	**+**	**+**	**+**	**-**	**-**	**-**	**-**	**-**	**-**

TSA plates were seeded with bacteria and 10 µl phage lysate (~10^9^ pfu/ml) spotted onto the plate prior to overnight 37°C.

Phage GBH014 and GBH038 were both able to lyse the six *K. pneumoniae* K2 isolates examined (**Table 2**). BLASTX revealed three potential K2 depolymerase candidate genes, GBH014_001 (215 residues) and GBH014_051 (433 residues) from phage GBH014 and GBH038_054 (577 residues) from phage GBH038. With GBH014_001, JACKHMMER showed matches to phage tailspikes and tail fibres, and to tailspike 63D sialidase from phage 1611E-K2-1, shown to be an active K2 depolymerase (Wang et al., 2020). In addition, a galactose binding domain (residues 65-214) was identified by InterProScan; galactose is a constituent of the K2 capsular polysaccharide (Clements et al., 2008). GBH014_051 showed matches to tail fibres using JACKHMMER and to serralysin metalloprotease using InterProScan. We were unable to purify and test this protein. GBH014_001 was cloned into pET26b+ both with and without a *pelB* tag on the N-terminus to migrate the protein to the periplasm and aid folding. GBH014_001 expressed better without the addition of the *pelB* tag, but could not be purified without denaturation of the protein. Refolded protein possessed no depolymerase activity when tested against K1, K2 and K51 capsules. GBH038_054 (577 residues) showed >98% amino acid homology with vP_KpnP_KpV74_564, the sole K2 depolymerase published to date (Solovieva et al., 2018) and was inserted between the HindIII and XhoI sites of pET26b+ with and without N-terminal *pelB* and grown for 16 h at 16°C following induction with 0.01 mM IPTG at OD_600_ ~0.4. The gene was expressed only in the presence of *pelB*. In spot tests, affinity-purified protein degraded the K2 capsule, but not K1, K51, K10 or K102 capsules (**Table 3**), or any of the capsules described in the preceding paragraph.

Only jumbo phage GBH019 and closely related phage were able to lyse the five *K. pneumoniae* K51 isolates examined (**Table 2**). Putative depolymerase genes were restricted to a 30,000 bp section of the GBH019 genome (125,000-155,000 bp) where eight CDSs (278-285) were located in series; such clustering of depolymerase genes is consistent with a recent study of jumbo *K. pneumoniae* phage phiK64-1 (Pan et al., 2017). There are currently no published K51 depolymerase sequences and none of the eight putative depolymerases showed sufficient homology with currently available sequences to guide the identification of a K51 depolymerase gene. Attempts were therefore made to clone and express these eight genes. Although all showed some degree of alignment with sequences in the protein database (**Supplementary Table 3**), the majority could not be expressed as enzymatically active protein. GBH019_279 (809 residues) showed limited alignment with tail fibre and tail spike proteins within the first 200 residues. This was supported by InterProScan analysis which predicted a pectin-lyase fold between residues 139 and 532, and Phyre2 modelling (Kelley et al., 2015) which predicted a hydrolase with 98.7% confidence, suggesting potential depolymerase activity but with no link to the specific K51 capsule target. BH019_279 was cloned into pET26b+ with *pelB*, the gene inserted between HindIII and XhoI sites, grown for 16 h at 16°C following induction with 0.01 mM IPTG at OD_600_ ~0.4 and the protein affinity-purified in soluble form. Spot tests indicated that this gene product degraded the K51 capsule but had no effect on any other capsule types in our K-type panel described above.

GBH001_056 (K1 depolymerase), GBH038_054 (K2 depolymerase) and GBH019_279 (K51 depolymerase) were assigned molecular weights of 71 kDa, 66 kDa and 93 kDa respectively using SDS-PAGE and were in line with values predicted from the size of the coding region of each gene.

### Characterisation of K1, K2 and K51 Depolymerases

**Figure 4:**
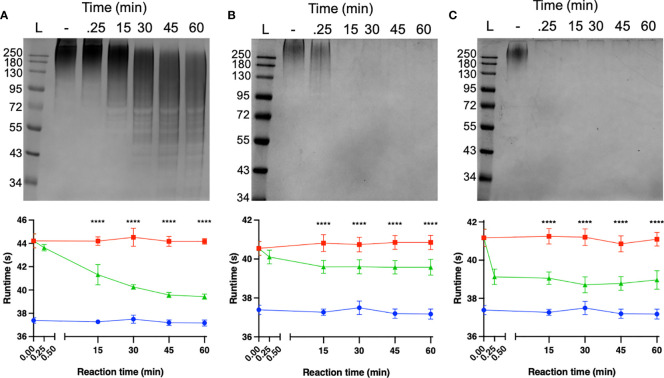
Degradation of high-molecular-weight capsular polysaccharide from *K*. *pneumoniae* SR65 **(A)**, SR3 **(B)** and TU16 **(C)** following incubation at 37°C with the corresponding K1, K2 and K51 depolymerases. 10% SDS-PAGE gels; 5 μl Color Prestained Protein Standard, Broad Range (10-250 kDa) (New England Biolabs UK) as ladder; 22.5 μl loaded into each lane. Enzyme-mediated reductions in polymer viscosity as determined by Anton-Paar rolling ball viscometry are also shown alongside the corresponding gels. No polymer degradation was observed in the absence of enzyme. ****P = < 0.0001 (unpaired t-test with Welch’s correction between treated and untreated groups).

**Figure 5:**
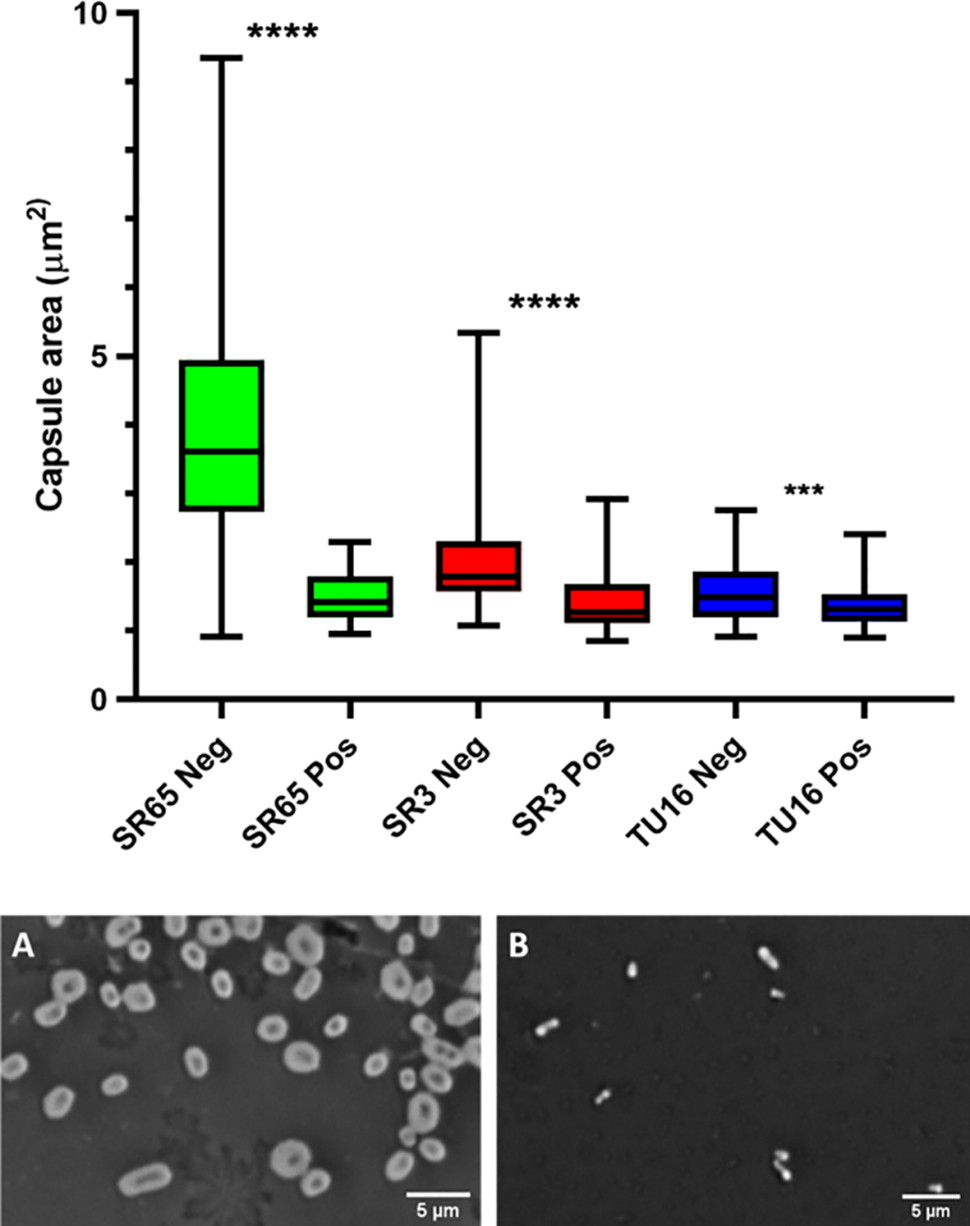
Box and whiskers plot of the impact on capsules of the target *K*. *pneumoniae* isolates SR65 (K1), SR3 (K2) and TU16 (K51) following 90 min incubation at 37°C with the corresponding K1, K2 and K51 depolymerases. SR65 Neg, negative control (PBS incubation), *n* = 319, mean capsule area μm^2^ 3.41 ± 1.00 (Mean ± 1SD); SR65 Pos, K1 depolymerase-exposed, *n* = 74, 1.49 ± 0.34; SR3 Neg, *n* = 119, 1.96 ± 0.58; SR3 Pos, *n* = 66, 1.41 ± 0.42; TU16 Neg, *n* = 84, 1.54 ± 0.42; TU16 Pos, *n* = 170, 1.36 ± 0.31. ****P = < 0.0001, ***P = < 0.002 (unpaired t-test with Welch’s correction between treated and untreated groups). Also shown: phase contrast microscopy images of nigrosin staining for isolate SR65 (K1) after 90 min incubation in PBS **(A)** and with K1 depolymerase **(B)**. Capsule can be seen in **(A)** as a bright halo around the cell.

The capacity of each depolymerase to degrade their respective primary substrates was determined by observing reductions in polysaccharide molecular mass and polysaccharide viscosity. Capsular substrates were purified from two K1 (SR7 & SR65), two K2 (SR3 & SR10) and two K51 (TU16 & SR54) clinical isolates. Essentially identical results were obtained for each K-type-specific pair so only data for the SR65, SR3 and TU16 polymers is presented in **Figure 4**. The three enzymes rapidly reduced the molecular mass with evidence of degradation after less than 1 min incubation time. After 60 min incubation each polymer appeared substantially degraded although depolymerisation of K2 and K51 polymers appeared greater than for K1. Reduction in molecular mass was accompanied in all cases by a large decrease in polymer viscosity using an Anton-Paar rolling ball viscometer. *K. pneumoniae* SR65 (K1), SR3 (K2) and TU16 (K51) were also employed to examine the capacity of the three depolymerases to remove the primary target capsule. The area occupied by the capsule was determined before addition of enzyme (final concentration 25 ng/µl for K1 depolymerase, 25 ng/µl for K2 depolymerase and 1.6 ng/µl for K51 depolymerase) and after 90 min incubation at 37°C following addition of the respective depolymerase. As noted in an earlier study (Loraine et al., 2018), there were significant differences in capsule size between each of the clinical isolates. K1, K2 and K51 depolymerases removed completely the capsules surrounding, respectively, isolates SR65, SR3 and TU16 (**Figure 5**). No significant differences were seen between bacteria prior to incubation and following 90 min incubation with PBS. Phase contrast images for *K. pneumoniae* SR65 are also shown in **Figure 5**.

## Discussion

The ideal candidate for capsule depolymerase therapy would be a difficult-to-treat infection caused by a single bacterial infectious agent producing a protective capsule that does not vary in its chemical composition between isolates and is essential for pathogenesis. Inhalation anthrax, due to *B. anthracis* strains that universally produce a poly-γ-D-glutamic acid capsule that is absolutely required for systemic, often lethal infection (Mock and Fouet, 2001), is probably the only infectious condition in which these tenets hold, so capsule depolymerisation as a principle for the therapy of other bacterial infections will be conditioned by a degree of structural variability, sometimes very large, at the cell surface. Our study confirms previous work showing that the host range of *K. pneumoniae* phage is to a large extent governed by carriage of distinct capsule depolymerases, each with a narrow substrate specificity (reviewed by Knecht et al., 2020). In a particularly illustrative example of this principal, the capacity of phage ΦK64-1 to infect *K. pneumoniae* belonging to eight capsule chemotypes was facilitated by carriage by the phage of eight capsule depolymerases, each capable of degrading only one structurally distinct capsule (Pan et al., 2017). Strains of *K. pneumoniae* elaborate a wide array of different capsule types (Wyres et al., 2020) and this population diversity was reflected in the large number of distinct K-types we found in our analysis of Thai nosocomial isolates (Loraine et al., 2018); only one K-type (K2) was found in more than 10% of total isolates and only six were found with an incidence of 4.5% or more. Successful application of depolymerase therapy will therefore require a cocktail of enzymes in much the same way as proposed from phage therapy of *Klebsiella* infections (Townsend et al., 2021), unless personalised therapy can be augmented in tandem with rapid and accurate strain identification and K-typing.

Although bacteria have been exploited as occasional sources of capsule depolymerase (Avery and Dubos, 1930; Scorpio et al., 2007; Stabler et al., 2013), phage remain the primary source of O-glycosyl hydrolases and lyases with the capacity to selectively degrade a wide range of capsules (Knecht et al., 2020), including those from *K. pneumoniae*, and for the dispersal of biofilms (Rumbaugh and Sauer, 2020). The capsular diversity of *K. pneumoniae* correlates to a correspondingly high degree of variation of phage genotypes that have evolved a lifestyle enabling lytic cycles within specific K-type hosts, with the consequence that we were able to isolate a diverse selection of phage, belonging to four families and displaying a wide range of genome sizes (41-348 kb), from hospital sewage using three different K-types as host. The majority of these phage were distinct from, but genomically related to, phage described in previous studies (**Figure 1B**). Plaques from all phage developed halos when plated on their primary and on the majority of secondary hosts, indicating the presence of a soluble form of capsule depolymerase that diffuses into the surrounding agar from the plaque during incubation. Genomic analyses were complicated by the presence of prophage sequences in a small number of DNA preparations that were presumably released from the host genome during phage lysate preparation as a consequence of host DNA damage, or prophage may have been induced by the lytic phage (Raya and Hebert, 2009; Townsend et al., 2021). It is however unlikely that this low level of contamination would have compromised the host range determined in this study (**Table 2** and **Supplementary Table 2**).

Phage-encoded capsule depolymerases are predominantly located in receptor binding proteins (RBPs) structured as tail fibres or tailspikes. *In situ*, these structures form trimers and each unit generally consists of a conserved N-terminal anchor domain that aids phage self-assembly, a K-type-variable central β-helical domain for host recognition and catalysis, and a C-terminal domain responsible for trimerisation and receptor recognition (Latka et al., 2017; Latka et al., 2019). Although direct evidence is lacking, it is generally accepted that both the soluble and integrated forms of RBP can freely diffuse through an agar matrix and that soluble and integrated monomers are structurally identical (Latka et al., 2017). However, due to their aggregative properties and multi-domain structure, RBPs are likely to form aqueous microparticulate dispersions rather than true solutions (Chi et al., 2003) and this may compromise high yield RBP expression. In the current study, as well as in our previous work (Leggate et al., 2002; Negus and Taylor, 2014), issues of solubility and low yield were encountered which in the current study were in part overcome by introduction of the *pelB* leader sequence into the plasmid vector, which directs the protein to the bacterial periplasm. Even so, a number of putative depolymerase genes identified in our screen could not be expressed as active protein in spite of repeated efforts: one possible way towards simplification of expression of a range of highly catalytic depolymerases could involve excision of the central catalytic domain of the RBP, where expression is more likely to lead to a fully soluble, enzymatically active polypeptide (Squeglia et al., 2020).

All seven exemplary phage that we studied in detail were closely related to other, previously described *Klebsiella* virions (**Figure 1B**), even though they were isolated from widely dispersed geographical regions. Of interest was the isolation of the jumbo phage GBH019, the source of the K51 depolymerase described herein, that counts amongst the largest *Klebsiella* phage isolated to date. In common with other jumbo phage, the GBH019 genome contained multiple putative depolymerase genes and this phage may be a source of enzymes for the degradation of the less abundant K-types that we encountered in our study of nosocomial Thai isolates (Loraine et al., 2018). Although relatively few jumbo phage have been isolated and characterised to date, direct sequencing of phage DNA from diverse ecosystems has revealed that they are highly abundant (Al-Shayeb et al., 2020) and as they are excluded from typical phage preparations processed using membrane filters (Yuan and Gao, 2017) they are unlikely to be recovered unless special precautions are employed during their isolation. In common with other jumbo phage, the GBH019 genome carries a large number of genes for *de novo* synthesis of purines and pyrimidines, DNA and RNA polymerases and interconversion of nucleotide phosphorylation states (**Figure 3**). As has been previously noted (Al-Shayeb et al., 2020) these gene sets are similar to those of small bacteria and archaea with restricted symbiotic lifestyles (Castelle et al., 2018), may reduce the dependence of jumbo phage on their bacterial hosts and broaden their host range by acquisition of new genetic information, as reflected by the presence of multiple depolymerase genes. The presence of CRISPR-Cas systems is also a feature of jumbo phage and may be involved in the redirection of bacterial host biosynthesis toward phage-encoded functions as well as contribute to host-directed elimination of incoming phage. Indeed, the first description of phage-encoded CRISPR-Cas showed the system was able to counteract a phage inhibitory chromosomal island of the bacterial host (Seed et al., 2013).

The increasing number of *Klebsiella* phage genomes in the public domain show certain trends in their genome sequences. Whereas most of the phage genomes in this study and in public databases have genomes with %GC of 50-55, similar to that of their bacterial host (56-57), jumbo phage genomes had markedly more AT–rich genomes with ~32% GC. A group of phage genomes, including that of phage GBH033 from this study, are also relatively AT-rich with GC% of ~40%. These two groups of phages are notable for their content of multiple tRNA genes that may serve to lessen the possible impact of divergent codon usages in their genomes when subverting their hosts’ metabolism. Comparative genomic analyses of our phage genomes with those in the databases revealed a number of phages that may represent novel phage species (with >5% sequence divergence and other differences) according to recent guidance from the ICTV (Adriaenssens et al., 2020). The increasing number of *Klebsiella* phage genomes deposited in databases increases our basic knowledge of gene composition and key phage phenotypic characteristics such as host range if appropriate metadata is available. However, difficulties in assigning functions to the majority of *Klebsiella* phage genes is an issue that is especially marked in jumbo phage genomes, where approximately three quarters of genes have no annotated function.

The trend toward untreatable, invasive *K. pneumoniae* infections shows no signs of abating and is being driven by the emergence and rapid spread of multi-drug-resistant forms. The problem is particularly acute in Asia, where hypervirulent *K. pneumoniae* clones associated with pyogenic liver abscesses, pneumonia, and meningitis in younger, otherwise healthy patients were first recognised, and as a consequence we have focused our efforts on finding alternative approaches to *Klebsiella* infections using recent clinical isolates from intensive care patients in Thailand (Loraine et al., 2018). There are clear advantages as well as obvious disadvantages in targeting capsule removal as an alternative therapeutic paradigm. Although there is substantial data from animal models showing that administration of capsule depolymerase under tightly controlled conditions during the early phase of infection with a known pathogen can rapidly resolve infection and prevent symptoms and death, there is no evidence that the approach would reduce morbidity and mortality in immunocompromised patients with established infections who are sick enough to be cared for in intensive care units. The complex array of surface-structure-related phenotypes of *K. pneumoniae* encountered in nosocomial infections will also complicate therapy. However, the capsule is the major determinant of virulence in *Klebsiella*, conferring a high degree of resistance to complement (Merino et al., 1992; Short et al., 2020) and to phagocytosis (Williams et al., 1983) and is rapidly and efficiently destroyed, *in vitro* and *in situ*, by each enzyme that we evaluated.

## Data Availability Statement

The datasets presented in this study can be found in online repositories. The names of the repository/repositories and accession number(s) can be found in the article/**Supplementary Material**.

## Author Contributions

PT conceived the study. GB-H, ME, NR, DN and PT designed experimental procedures. GB-H, ME, DN, DP, GB, MD and PW performed the experiments, analysed and curated the data. SV, PW and GB-H assembled the phage collection. PT and ME wrote the manuscript. All authors contributed to the article and approved the submitted version.

## Funding

The study was funded by the UCL Therapeutic Acceleration Support Fund (supported by the Medical Research Council [MRC] Confidence in Concept and Wellcome Trust Institutional Strategic Support Funds), MRC project grant MR/R009937/1, the Newton Fund through MRC award MR/N012542/1 and National Science and Technology Development Agency (NSTDA) award FDA-CO2559-1448-TH and Wellcome Trust Collaborative Award 201505/Z/16/Z. MJD is a Junior Research Fellow at Churchill College, Cambridge and his research is funded in whole, or in part, by Wellcome Trust grant 206194.

## Conflict of Interest

The authors declare that the research was conducted in the absence of any commercial or financial relationships that could be construed as a potential conflict of interest.

## Supplementary Material

Supplementary Figure 1 Genomic comparison of four *K. pneumoniae* jumbo phage: Muenster (top), GBH019, K64-1, and vB_KleM-RaK2 (bottom) highlighting regions of gene similarity and synteny. Red bars indicate >99% BLASTN similarity between pairs of phage strains. Generated using Artemis Comparison Tool (Carver et al., 2005) with pairwise BLASTN comparisons using default parameters for highly similar sequences.

Supplementary Table 1 Primers and conditions for cloning of depolymerase genes.

Supplementary Table 2 Host range of 62 K*. pneumoniae* phage isolated from Thai sewage.

Supplementary Table 3 Published protein sequences of phage-encoded *K. pneumoniae* depolymerases employed for BLASTX search of Thai *K. pneumoniae* phage genomes.

Supplementary Table 4 Accession numbers for sequenced phage. Formal numbering of phage is affirmed in this table.

Supplementary Data Sheet 1 Sequences of plasmids used in this study.
