# Supplementary figures and images for: Characterisation of Bacteriophage-Encoded Depolymerases Selective for Key Klebsiella pneumoniae Capsular Exopolysaccharides
Source: Front Cell Infect Microbiol. 2021 Jun 18;11:686090. doi: 10.3389/fcimb.2021.686090 (PMC8253255; doi:10.3389/fcimb.2021.686090)

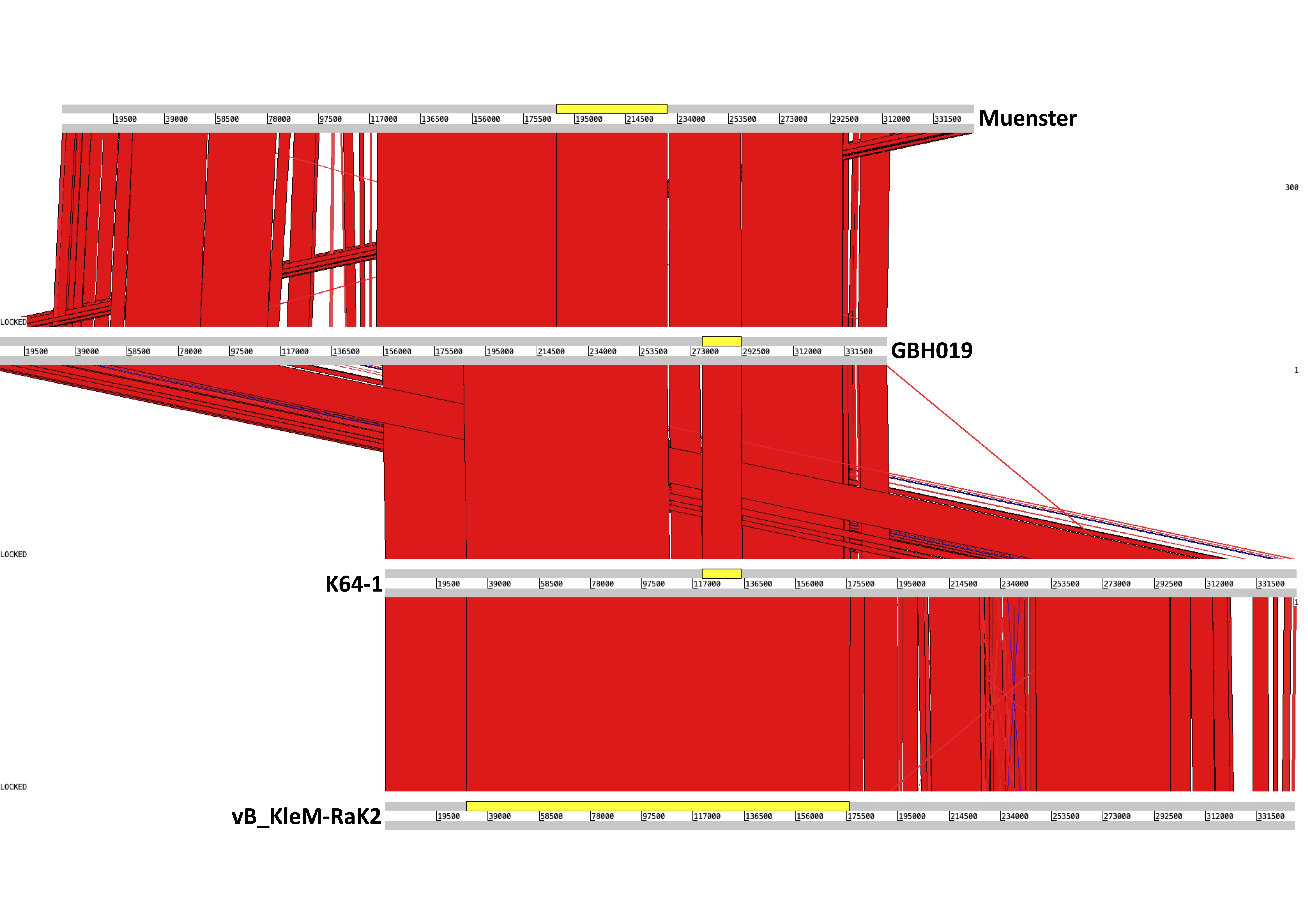

Supplement: Supplementary Figure 1 — Genomic comparison of four K. pneumoniae jumbo phage: Muenster (top), GBH019, K64-1, and vB_KleM-RaK2 (bottom) highlighting regions of gene similarity and synteny. Red bars indicate >99% BLASTN similarity between pairs of phage strains. Generated using Artemis Comparison Tool (Carver et al., 2005) with pairwise BLASTN comparisons using default parameters for highly similar sequences. [file Image_1.jpeg]
