# Supplementary material for: Characterisation of Bacteriophage-Encoded Depolymerases Selective for Key Klebsiella pneumoniae Capsular Exopolysaccharides
Source: Front Cell Infect Microbiol. 2021 Jun 18;11:686090. doi: 10.3389/fcimb.2021.686090 (PMC8253255; doi:10.3389/fcimb.2021.686090)
Supplement: Supplementary Table 1 — Primers and conditions for cloning of depolymerase genes. [file Table_1.docx]

| Primer name | Primer sequence | Annealing temperature | Extension time |
| --- | --- | --- | --- |
| GBH001_048 F (NdeI) | gatcCATATGGCATACAGCTGGCA | 65 | 60s |
| GBH001_048 R (XhoI) | gatcCTCGAGTCTTACCCTCATAGCTCTAATATAACCC | 65 | 60s |
| GBH001_056 F (NdeI) | gatcCATATGGCATTAATTAGATTAGTAGCTCC | 62 | 60s |
| GBH001_056 R (XhoI) | gatcCTCGAGGAGATACACCTCCCAGG | 62 | 60s |
| GBH014_001 F (NdeI) | gatcCATATGGTGTTTGTTGGCGCAAAG | 62 | 45s |
| GBH014_001 R (XhoI) | gatcCTCGAGCAAGAAGTTTACGATAACGTCATC | 62 | 45s |
| GBH038_054 F (HindIII) | gatcAAGCTTATGGCACTAGTAGATTTAGTGAG | 62 | 60s |
| GBH038_054 R (XhoI) | gatcCTCGAGCTTGACCACGTTGCAC | 62 | 60s |
| GBH019_279 F (HindIII) | gatcAAGCTTATGGCGAATGAATTAATCCAAC | 60 | 90s |
| GBH019_279 R (XhoI) | gatcCTCGAGAGAAGATATGGAACCATAAGTTAG | 60 | 90s |

**TABLE S1** Primers and conditions for cloning of depolymerase genes
