# Supplementary material for: Characterisation of Bacteriophage-Encoded Depolymerases Selective for Key Klebsiella pneumoniae Capsular Exopolysaccharides
Source: Front Cell Infect Microbiol. 2021 Jun 18;11:686090. doi: 10.3389/fcimb.2021.686090 (PMC8253255; doi:10.3389/fcimb.2021.686090)
Supplement: Supplementary Table 2 — Host range of 62 K. pneumoniae phage isolated from Thai sewage. [file Table_2.docx]

|  |  | **Strain/isolate; capsule (K) type** | | | | | | | | | | | | | | | | | | | | |  |
| --- | --- | --- | --- | --- | --- | --- | --- | --- | --- | --- | --- | --- | --- | --- | --- | --- | --- | --- | --- | --- | --- | --- | --- |
|  |  | **SR7** | **SR65** | **TU37** | **NTUH-**  **K2044** | **SR3** | **TU18** | **SG44** | **SR10** | **SR51** | **ATCC-**  **43816** | **SR57** | **TU16** | **SG45** | **SR54** | **TU9** | **TU29** | **SR4** | **SG95** | **TU1** | **SG41** | **SG56** | **WGS^c^** |
| **Phage** | **Isolation**^a^ | **K1** | | | | **K2** | | | | | | **K51** | | | | | **K10** | | | **K102** | | |  |
| **GBH001** | **TU37** | **++**^b^ | **+** | **++** | **++** | **-** | **-** | **-** | **+** | **-** | **-** | **-** | **+** | **-** | **-** | **-** | **-** | **-** | **-** | **-** | **-** | **-** | **🗸** |
| **GBH002** | **TU37** | **++** | **+** | **++** | **++** | **-** | **-** | **-** | **-** | **-** | **-** | **-** | **+** | **-** | **-** | **-** | **-** | **-** | **-** | **-** | **-** | **-** | **🗸** |
| **GBH003** | **TU37** | **++** | **+** | **++** | **+** | **-** | **-** | **-** | **-** | **-** | **-** | **-** | **+** | **-** | **-** | **+** | **-** | **-** | **-** | **-** | **-** | **-** | **🗸** |
| **GBH004** | **TU37** | **++** | **+** | **++** | **++** | **-** | **-** | **-** | **-** | **-** | **-** | **-** | **+** | **-** | **-** | **-** | **-** | **-** | **-** | **-** | **-** | **-** |  |
| **GBH005** | **TU37** | **++** | **+** | **++** | **++** | **-** | **-** | **-** | **-** | **-** | **-** | **-** | **+** | **-** | **-** | **-** | **-** | **-** | **-** | **-** | **-** | **-** | **🗸** |
| **GBH006** | **TU37** | **++** | **+** | **++** | **++** | **-** | **-** | **-** | **-** | **-** | **-** | **-** | **-** | **-** | **-** | **-** | **-** | **-** | **-** | **-** | **-** | **-** |  |
| **GBH007** | **TU37** | **++** | **+** | **++** | **++** | **-** | **-** | **+** | **++** | **-** | **-** | **-** | **+** | **-** | **-** | **-** | **-** | **-** | **-** | **-** | **-** | **-** | **🗸** |
| **GBH008** | **TU37** | **_++_** | **+** | **++** | **++** | **-** | **-** | **-** | **-** | **-** | **-** | **-** | **-** | **-** | **-** | **-** | **-** | **-** | **-** | **-** | **-** | **-** |  |
| **GBH009** | **TU37** | **++** | **+** | **++** | **++** | **-** | **-** | **-** | **-** | **-** | **-** | **-** | **-** | **-** | **-** | **-** | **-** | **-** | **-** | **-** | **-** | **-** |  |
| **GBH010** | **TU37** | **++** | **+** | **++** | **++** | **-** | **-** | **-** | **-** | **-** | **-** | **-** | **-** | **-** | **-** | **-** | **-** | **-** | **-** | **-** | **-** | **-** |  |
| **GBH011** | **TU37** | **++** | **+** | **++** | **++** | **-** | **-** | **+** | **-** | **-** | **-** | **-** | **-** | **-** | **-** | **-** | **-** | **-** | **-** | **-** | **-** | **-** |  |
| **GBH012** | **TU37** | **++** | **+** | **++** | **++** | **-** | **-** | **-** | **-** | **-** | **-** | **-** | **-** | **-** | **-** | **-** | **-** | **-** | **-** | **-** | **-** | **-** |  |
| **GBH013** | **SG44** | **++** | **++** | **++** | **++** | **++** | **++** | **++** | **++** | **++** | **++** | **-** | **+** | **-** | **-** | **-** | **-** | **-** | **-** | **-** | **-** | **-** | **🗸** |
| **GBH014** | **SG44** | **+** | **-** | **-** | **-** | **++** | **++** | **++** | **++** | **++** | **++** | **-** | **-** | **-** | **-** | **-** | **-** | **-** | **-** | **-** | **-** | **-** | **🗸** |
| **GBH015** | **SG44** | **-** | **-** | **-** | **-** | **++** | **++** | **++** | **++** | **++** | **++** | **-** | **+** | **-** | **-** | **-** | **-** | **-** | **-** | **-** | **-** | **-** |  |
| **GBH016** | **SG44** | **-** | **-** | **-** | **-** | **++** | **++** | **++** | **++** | **++** | **++** | **-** | **-** | **-** | **-** | **-** | **-** | **-** | **-** | **-** | **-** | **-** |  |
| **GBH017** | **SG44** | **++** | **++** | **++** | **++** | **++** | **++** | **++** | **++** | **++** | **++** | **-** | **+** | **-** | **-** | **-** | **-** | **-** | **-** | **-** | **-** | **-** | **🗸** |
| **GBH018** | **SG44** | **-** | **-** | **-** | **-** | **++** | **++** | **++** | **++** | **-** | **++** | **-** | **++** | **-** | **-** | **-** | **-** | **-** | **-** | **-** | **+** | **-** | **🗸** |
| **GBH019** | **TU9** | **-** | **-** | **-** | **-** | **-** | **-** | **+** | **-** | **-** | **-** | **++** | **++** | **++** | **++** | **++** | **-** | **-** | **-** | **+** | **++** | **++** | **🗸** |
| **GBH020** | **TU9** | **-** | **-** | **-** | **-** | **-** | **-** | **-** | **-** | **-** | **-** | **++** | **++** | **++** | **++** | **++** | **-** | **-** | **-** | **+** | **++** | **++** | **🗸** |
| **GBH021** | **TU9** | **-** | **-** | **-** | **-** | **-** | **-** | **-** | **-** | **-** | **-** | **++** | **++** | **++** | **++** | **++** | **-** | **-** | **-** | **+** | **++** | **++** |  |
| **GBH022** | **TU9** | **-** | **-** | **-** | **-** | **-** | **-** | **-** | **-** | **-** | **-** | **++** | **++** | **++** | **++** | **++** | **-** | **-** | **-** | **+** | **++** | **++** |  |
| **GBH023** | **TU9** | **-** | **-** | **-** | **-** | **-** | **-** | **-** | **-** | **-** | **-** | **++** | **++** | **++** | **++** | **++** | **-** | **-** | **-** | **+** | **++** | **++** | **🗸** |
| **GBH024** | **TU9** | **-** | **-** | **-** | **-** | **-** | **-** | **-** | **-** | **-** | **-** | **++** | **++** | **++** | **++** | **++** | **-** | **-** | **-** | **+** | **++** | **++** |  |
| **GBH025** | **SG43** | **-** | **-** | **-** | **-** | **-** | **-** | **++** | **+** | **-** | **++** | **-** | **++** | **-** | **+** | **+** | **-** | **+** | **-** | **-** | **+** | **-** |  |
| **GBH026** | **SG43** | **-** | **-** | **-** | **-** | **-** | **++** | **++** | **+** | **-** | **++** | **-** | **++** | **-** | **+** | **+** | **-** | **+** | **-** | **-** | **+** | **-** | **🗸** |
| **GBH027** | **SG43** | **-** | **-** | **-** | **-** | **-** | **++** | **++** | **+** | **-** | **+** | **-** | **++** | **-** | **+** | **+** | **-** | **+** | **-** | **-** | **+** | **-** | **🗸** |
| **GBH028** | **SG43** | **-** | **-** | **-** | **-** | **-** | **-** | **++** | **+** | **-** | **+** | **-** | **++** | **-** | **-** | **-** | **-** | **+** | **-** | **-** | **-** | **-** |  |
| **GBH029** | **SG43** | **-** | **-** | **-** | **-** | **-** | **-** | **++** | **+** | **-** | **+** | **-** | **++** | **-** | **-** | **-** | **-** | **+** | **-** | **-** | **-** | **-** | **🗸** |
| **GBH030** | **SG45** | **-** | **-** | **-** | **-** | **-** | **-** | **++** | **+** | **-** | **+** | **-** | **++** | **-** | **-** | **-** | **-** | **+** | **-** | **-** | **-** | **-** |  |
| **GBH031** | **SG45** | **-** | **-** | **-** | **-** | **-** | **-** | **-** | **-** | **-** | **-** | **-** | **++** | **-** | **-** | **-** | **-** | **-** | **-** | **-** | **-** | **-** |  |
| **GBH032** | **SG45** | **-** | **-** | **-** | **-** | **-** | **-** | **-** | **-** | **-** | **-** | **-** | **++** | **-** | **-** | **-** | **-** | **-** | **-** | **-** | **-** | **-** |  |
| **GBH033** | **SG45** | **-** | **-** | **-** | **-** | **-** | **-** | **+** | **-** | **-** | **+** | **-** | **++** | **+** | **+** | **-** | **-** | **-** | **-** | **-** | **-** | **-** | **🗸** |
| **GBH034** | **SG45** | **-** | **-** | **-** | **-** | **-** | **-** | **+** | **-** | **-** | **-** | **-** | **++** | **+** | **+** | **-** | **-** | **-** | **-** | **-** | **-** | **-** |  |
| **GBH035** | **SG45** | **-** | **-** | **-** | **-** | **-** | **-** | **++** | **+** | **-** | **+** | **-** | **++** | **+** | **+** | **-** | **-** | **-** | **-** | **-** | **-** | **-** | **🗸** |
| **GBH036** | **SG46** | **-** | **-** | **-** | **-** | **+** | **++** | **++** | **++** | **++** | **++** | **-** | **-** | **-** | **-** | **-** | **-** | **-** | **-** | **-** | **-** | **-** |  |
| **GBH037** | **SG46** | **-** | **-** | **-** | **-** | **++** | **++** | **++** | **++** | **++** | **++** | **-** | **-** | **-** | **-** | **-** | **-** | **-** | **-** | **-** | **-** | **-** |  |
| **GBH038** | **SG46** | **-** | **-** | **-** | **-** | **++** | **++** | **++** | **++** | **++** | **++** | **-** | **-** | **-** | **-** | **-** | **-** | **-** | **-** | **-** | **-** | **-** | **🗸** |
| **GBH039** | **SG46** | **-** | **-** | **-** | **-** | **++** | **++** | **++** | **++** | **++** | **++** | **-** | **-** | **-** | **-** | **-** | **-** | **-** | **-** | **-** | **-** | **-** | **🗸** |
| **GBH040** | **SG46** | **-** | **-** | **-** | **-** | **++** | **++** | **++** | **++** | **++** | **++** | **-** | **-** | **-** | **-** | **-** | **-** | **-** | **-** | **-** | **-** | **-** |  |
| **GBH041** | **SG46** | **-** | **-** | **-** | **-** | **++** | **++** | **++** | **++** | **++** | **++** | **-** | **-** | **-** | **-** | **-** | **-** | **-** | **-** | **-** | **-** | **-** |  |
| **GBH042** | **TU18** | **-** | **-** | **-** | **-** | **++** | **++** | **++** | **++** | **++** | **++** | **-** | **-** | **-** | **-** | **-** | **-** | **-** | **-** | **-** | **-** | **-** |  |
| **GBH043** | **TU18** | **-** | **-** | **-** | **-** | **++** | **++** | **++** | **++** | **++** | **++** | **-** | **-** | **-** | **-** | **-** | **-** | **-** | **-** | **-** | **-** | **-** |  |
| **GBH044** | **TU18** | **-** | **-** | **-** | **-** | **++** | **++** | **++** | **++** | **++** | **++** | **-** | **-** | **-** | **-** | **-** | **-** | **-** | **-** | **-** | **-** | **-** |  |
| **GBH045** | **TU18** | **-** | **-** | **-** | **-** | **++** | **++** | **++** | **++** | **++** | **++** | **-** | **-** | **-** | **-** | **-** | **-** | **-** | **-** | **-** | **-** | **-** | **🗸** |
| **GBH046** | **TU18** | **-** | **-** | **-** | **-** | **++** | **++** | **++** | **++** | **++** | **++** | **-** | **-** | **-** | **-** | **-** | **-** | **-** | **-** | **-** | **-** | **-** | **🗸** |
| **GBH047** | **TU18** | **-** | **-** | **-** | **-** | **++** | **++** | **++** | **++** | **++** | **++** | **-** | **-** | **-** | **-** | **-** | **-** | **-** | **-** | **-** | **-** | **-** |  |
| **GBH048** | **TU30** | **-** | **-** | **-** | **-** | **++** | **++** | **++** | **++** | **++** | **++** | **-** | **-** | **-** | **-** | **-** | **-** | **-** | **-** | **-** | **-** | **-** |  |
| **GBH049** | **TU30** | **-** | **-** | **-** | **-** | **++** | **++** | **++** | **++** | **++** | **++** | **-** | **-** | **-** | **-** | **-** | **-** | **-** | **-** | **-** | **-** | **-** | **🗸** |
| **GBH050** | **TU30** | **-** | **-** | **-** | **-** | **++** | **++** | **++** | **++** | **++** | **++** | **-** | **-** | **-** | **-** | **-** | **-** | **-** | **-** | **-** | **-** | **-** | **🗸** |
| **GBH051** | **TU30** | **-** | **-** | **-** | **-** | **++** | **++** | **++** | **++** | **++** | **++** | **-** | **-** | **-** | **-** | **-** | **-** | **-** | **-** | **-** | **-** | **-** |  |
| **GBH052** | **TU30** | **-** | **-** | **-** | **-** | **++** | **++** | **++** | **++** | **++** | **++** | **-** | **-** | **-** | **-** | **-** | **-** | **-** | **-** | **-** | **-** | **-** |  |
| **GBH053** | **TU30** | ^+^ | **-** | **-** | **-** | **++** | **++** | **++** | **++** | **++** | **++** | **-** | **-** | **-** | **-** | **-** | **-** | **-** | **-** | **-** | **-** | **-** |  |
| **GBH054** | **SG79** | **-** | **-** | **-** | **-** | **-** | **++** | **++** | **++** | **+** | **-** | **-** | **++** | **++** | **-** | **++** | **-** | **-** | **-** | **-** | **-** | **-** | **🗸** |
| **GBH055** | **SG79** | **-** | **-** | **-** | **-** | **-** | **++** | **++** | **++** | **-** | **-** | **+** | **++** | **++** | **+** | **++** | **-** | **-** | **-** | **-** | **-** | **-** | **🗸** |
| **GBH056** | **SG79** | **-** | **-** | **-** | **-** | **-** | **++** | **++** | **++** | **-** | **-** | **+** | **++** | **+** | **-** | **++** | **-** | **-** | **-** | **-** | **-** | **-** | **🗸** |
| **GBH057** | **SG79** | **-** | **-** | **-** | **-** | **-** | **-** | **++** | **++** | **-** | **-** | **-** | **++** | **++** | **-** | **++** | **-** | **-** | **-** | **-** | **-** | **-** |  |
| **GBH058** | **SG79** | **-** | **-** | **-** | **-** | **-** | **-** | **++** | **++** | **-** | **-** | **-** | **++** | **++** | **-** | **++** | **-** | **-** | **-** | **-** | **-** | **-** |  |
| **GBH059** | **SG79** | **-** | **-** | **-** | **-** | **-** | **-** | **+** | **++** | **-** | **-** | **+** | **++** | **++** | **+** | **++** | **-** | **-** | **-** | **-** | **-** | **-** |  |
| **GBH060** | **SG79** | **-** | **-** | **-** | **-** | **-** | **-** | **++** | **++** | **-** | **-** | **+** | **++** | **++** | **-** | **++** | **-** | **+** | **-** | **-** | **-** | **-** | **🗸** |
| **GBH061** | **SG79** | **-** | **-** | **-** | **-** | **-** | **-** | **+** | **++** | **-** | **-** | **++** | **++** | **++** | **+** | **++** | **-** | **-** | **-** | **-** | **-** | **-** | **🗸** |
| **GBH062** | **SG79** | **-** | **-** | **-** | **-** | **-** | **-** | **+** | **++** | **-** | **-** | **+** | **++** | **++** | **+** | **+** | **-** | **-** | **-** | **-** | **-** | **-** |  |

^a^clinical isolates are detailed in Table 1; ^b^clear plaque, **++**; **+**, incomplete lysis; **-,** no lysis. Determined by spot assay, phage concentration ~10^10^ virions/mL; ^c^WGS, phage selected for whole-genome sequencing

**TABLE S2** Host range of 62 *Klebsiella pneumoniae* phage isolated from Thai sewage
