# Supplementary material for: Characterisation of Bacteriophage-Encoded Depolymerases Selective for Key Klebsiella pneumoniae Capsular Exopolysaccharides
Source: Front Cell Infect Microbiol. 2021 Jun 18;11:686090. doi: 10.3389/fcimb.2021.686090 (PMC8253255; doi:10.3389/fcimb.2021.686090)
Supplement: Supplementary Table 3 — Published protein sequences of phage-encoded K. pneumoniae depolymerases employed for BLASTX search of Thai K. pneumoniae phage genomes. [file Table_3.docx]

| **Depolymerase** | **Capsule target** | **Accession number** | **Coverage, % identity with GBH001_056** | **Coverage, identity with GBH038_054** | **Coverage, identity with GBH019_279** |
| --- | --- | --- | --- | --- | --- |
| phiK64-1_S1-1^1^ | K11 | YP_009153197.1 |  | 48%, 26.38% | 14%, 59.13% |
| phiK64-1_S1-2^1^ | KN4 | YP_009153195.1 |  |  | 14%, 39.67% |
| phiK64-1_S1-3^1^ | K21 | YP_009153196.1 |  |  | 125, 30.77% |
| phiK64-1_S2-1^1^ | KN5 | YP_009153198.1 |  |  | 8%, 31.17% |
| phiK64-1_S2-2^1^ | K25 | YP_009153199.1 |  |  | 15%, 35.96% |
| phiK64-1_S2-3^1^ | K35 | YP_009153200.1 |  |  | 10%, 47.19% |
| phiK64-1_S2-4^1^ | K1 | YP_009153201.1 | 98%, 32.98% |  |  |
| phiK64-1_S2-5^1^ | K64 | YP_009153202.1 |  |  | 4%, 31.58% |
| phiK64-1_S2-6^1^ | K30, K69 | YP_009153203.1 | 5%, 34.21% |  | 7%, 35.38% |
| K5-2_ORF37^2^ | K30, K69 | APZ82804.1 |  |  |  |
| K5-4_ORF37^2^ | K8 | APZ82847.1 |  |  | 4%, 50% |
| K5-4_ORF38^2^ | K5 | APZ82848.1 | 13%, 25.71% |  |  |
| vB_KpnP_IME321_ORF42^3^ | KN1 | AXE28435.1 |  |  |  |
| vB_KpnP_KpV71_52^4^ | K1 | AMQ66478.1 | 99%, 95.24% | 15%, 31.11% |  |
| vB_KpnP_KpV74_56^4^ | K2 | APZ82768.1 | 11%, 31.52% | 99%, 98.61% |  |
| KP32_ORF37^5^ | K3 | YP_003347555.1 |  |  | 7%, 34.38% |
| KP32_ORF38^5^ | K21 | YP_003347556.1 |  |  |  |
| KN1-1_KN1dep^6^ | KN1 | BBF66844.1 |  |  |  |
| KN3-1_KN3dep^6^ | KN3 | BBF66867.1 |  |  | 8%, 30.99% |
| KN3-1_K56dep^6^ | K56 | BBF66868.1 |  |  |  |
| KN4-1_KN4dep^6^ | KN4 | BBF66888.1 |  |  |  |
| NTUH-K2044-K1-1_ORF34^7^ | K1 | YP_009098385.1 | 99%, 96.16% | 15%, 30.43% | 7%, 25.42% |
| 0507KN21_ORF96^8^ | KN2 | BAN78446.1 |  |  |  |
| SH-KP152226_Dep42^9^ | K47 | QDF14644.1 |  |  | 8%, 28.4% |
| KP36_depoKP36^10^ | K63 | YP_009226010.1 |  |  |  |

**TABLE S3** Published (up to December 2020) protein sequences of phage-encoded *K. pneumoniae* depolymerases employed for BLASTX search of Thai *K. pneumoniae* phage genomes. Superscript in **Depolymerase** column links to source reference as follows:

1. Pan, Y.J., Lin, T.L., Chen, C.C., Tsai, Y.T., Cheng, Y.H., Chen, Y.Y., et al. (2017). *Klebsiella* phage ΦK64-1 encodes multiple depolymerases for multiple host capsular types. J. Virol. 91:e02457-16. doi: 10.1128/JVI.02457-16
2. Hsieh, P.F., Lin, H.H., Lin, T.L., Chen, Y.Y., and Wang, J.T. Two T7-like bacteriophages, K5-2 and K5-4, each encodes two capsule depolymerases: isolation and functional characterization. (2017). Sci. Rep. 7:4624. doi: 10.1038/s41598-017-04644-2
3. Wang, C., Li, P., Niu, W., Yuan, X., Liu, H., Huang, Y., et al. (2019). Protective and therapeutic application of the depolymerase derived from a novel KN1 genotype of *Klebsiella pneumoniae* bacteriophage in mice. Res. Microbiol. 170:156-164. doi: 10.1016/j.resmic.2019.01.003
4. Solovieva, E.V., Myakinina, V.P., Kislichkina, A.A., Krasilnikova, V.M., Verevkin, V.V., Mochalov, V.V., et al. (2018). Comparative genome analysis of novel Podoviruses lytic for hypermucoviscous *Klebsiella pneumoniae* of K1, K2, and K57 capsular types. Virus Res. 243:10-18. doi: 10.1016/j.virusres.2017.09.026
5. Majkowska-Skrobek, G., Latka, A., Berisio, R., Squeglia, F., Maciejewska, B., Briers, Y., et al. (2018). Phage-borne depolymerases decrease *Klebsiella pneumoniae* resistance to innate defense mechanisms. Front. Microbiol. 9:2517. doi: 10.3389/fmicb.2018.02517.
6. Pan, Y.J., Lin, T.L., Chen, Y.Y., Lai, P.H., Tsai, Y.T., Hsu, C.R., et al. (2019). Identification of three podoviruses infecting *Klebsiella* encoding capsule depolymerases that digest specific capsular types. Microb. Biotechnol. 12:472-486. doi: 10.1111/1751-7915.13370
7. Lin, T.L., Hsieh, P.F., Huang, Y.T., Lee, W.C., Tsai, Y.T., Su, P.A., et al. (2014). Isolation of a bacteriophage and its depolymerase specific for K1 capsule of *Klebsiella pneumoniae*: implication in typing and treatment. J. Infect. Dis. 210:1734-1744. doi: 10.1093/infdis/jiu332
8. Hsu, C.R., Lin, T.L., Pan, Y.J., Hsieh, P.F., and Wang, J. T. (2013). Isolation of a bacteriophage specific for a new capsular type of *Klebsiella pneumoniae* and characterization of its polysaccharide depolymerase. PloS One. 8:e70092. [doi.org/10.1371/journal.pone.0070092](https://doi.org/10.1371/journal.pone.0070092)
9. Wu, Y., Wang, R., Xu, M., Liu, Y., Zhu, X., Qiu, J., et al. (2019). A novel polysaccharide depolymerase encoded by the phage SH-KP152226 confers specific activity against multidrug-resistant *Klebsiella pneumoniae via* biofilm degradation. Front. Microbiol. 10:2768. doi: 10.3389/fmicb.2019.02768
10. Majkowska-Skrobek, G., Łątka, A., Berisio, R., Maciejewska, B., Squeglia, F., Romano, M., et al. (2016). Capsule-targeting depolymerase, derived from *Klebsiella* KP36 phage, as a tool for the development of anti-virulent strategy. Viruses. 8:324. doi: 10.3390/v8120324
