# Supplementary material for: Characterisation of Bacteriophage-Encoded Depolymerases Selective for Key Klebsiella pneumoniae Capsular Exopolysaccharides
Source: Front Cell Infect Microbiol. 2021 Jun 18;11:686090. doi: 10.3389/fcimb.2021.686090 (PMC8253255; doi:10.3389/fcimb.2021.686090)
Supplement: Supplementary Data Sheet 1 — Sequences of plasmids used in this study. [file DataSheet_1.docx]

Plasmid sequence information:

DNA sequence from the bacteriophage genomes shown in uppercase letters. ORF containing the depolymerase and any tags shown in green.

GBH001_056_pET26b+:

atataggcgccagcaaccgcacctgtggcgccggtgatgccggccacgatgcgtccggcgtagaggatcgagatctcgatcccgcgaaattaatacgactcactataggggaattgtgagcggataacaattcccctctagaaataattttgtttaactttaagaaggagatatacaTATGGCATTAATTAGATTAGTAGCTCCCGAGCGGGTGTTCTCCGACTTGGCGAGCATGGTAGCATACCCAAACTTTCAGGTGCAGGACAAGATTATCCTACTGGGCAGTGGTGGTGGGGATTTCACCTTTACTACTACTGCGTCGGTAGTGGATAACGGAACTGTGTTTGCTGTACCCGGTGGGTACCTGCTCCGTAAATTCGTGGGCCCAGCATACAGCTCCTGGTTCAGCAACTGGGCGGGTATAGTCACGTTCATGAGTGCGCCTAATAGGCACCTGGTTGTGGACACGGTCCTGCAGGCCACGAGTGTGCTCAACATCAAAAGCAACTCTACGCTAGAGTTTACCGATACCGGAAGAATCCTACCGGATGCTGCGGTTGCACGTCAAGTGCTTAATATTATCGGCTCTGCGCCCTCGGCGTTCGTGCCGTTAGCAGCGGATGCCGCTGCAGGTAGCAAAGTCATTACGGTGACTGCTGGGGCTTTGTCTGTGGTAAAAGGTACGTACTTGTATCTTCGCTCTAACAAGCTGTGCGACGGTGGGCCTAACACGCACGGGGTAAAGATTTCCCAGATTAGGAAAGTGGTGGGTGTTAGCACCTCCGGTAGCATCACCAGCATTCGGCTGGATAAAGCGCTGCACTATAACTACTACCTGTCTGATGCCGCGGAAGTAGGTATCCCGACAATGGTGGAGAACGTAACCTTAGTATCCCCGTACATCAACGAGTTCGGCTACGACGACTTGAACCGGTTCTTTACTATCGGTATCTCTGCCAACTTTGCCGCGGACTTGCACATCCAGGATGGGGTTATTATTGGCAACAAACGCCCCGGGGCTTCTGATATAGAAGGGCGTAGTGCTATCAAGTTCAATAACTGCGTAGACAGCACCGTTAAGGGTACGTACTTCTACAACATCGGATGGTACGGGGTAGAGGTGATCGGCTGCTCAGAGGACACGGAAGTACATGATATCCACGCTATGGACGTACGCCACGCAATCTCTCTGAACTGGCAGAGCACTGCAGACGGGGACAAGTGGGGAGAGCCTATCGAGTTCTTAGGCGTTAACTGCGAAGCCTACAGCACAACCCATGCTGGATTTGATACTCACGACATTGGTAAGCGTGTGAAGTTCGTTCGCTGCGTTTCATACGACAGTGCTGACGATGGGTTCCAAGCCCGCACTAATGGGGTAGAGTACTTGAACTGTCGAGCTTACCGCGCCGCCCTAGACGGATTCGCCTCCAGCACCGGCGTAGGCTTCCCTATCTATAGAGAATGCTTAGCTTATGACAACGTACGCTCTGGATTTAACTGTTCGTACGGTGGTGGCTACGTTTATGACTGTGAGGCTCACGGTAGCCGGAATGGTGTCCGCACCAACGGGGGTTGTGTTAAGGGTGGTCGGTACACTCGCAACTCCAGCTCGCACATCTTCGTCACTAAGGATGTAGCTGAGACAGCGCAGACGTCCCTGGAGATTGATGGCGTGAGCATGCGCTACGATGGTACCGGAAGAGCTGTGTACTTCCACGGGACTATGGGCATTAACCCTACCCTGGTATCTATGTCCAATAATGACATGACCGGTCACGGCTTGTCCTGGGCATTACTGAGTGGCTACACTGTTCAACCTACACCCCCGCGCATGTCCAGGAACTTACTGGACGACACAAGCATTCGCGGAGTGGCAACGCTGGTTGCTGGTGAGGCTACAGTCAACGCCCGTGTACGCGGAAACTTTGGCAGTGTAGCTAACTCCTTCAAGTGGGTGTCCGAGGTTAAGTTGACGCGGTTGACCTTTCCCTCTAGTGCTGGGGCCTTGACGGTTACTAGCGTAGCTCAGAACCGGGATGTGCCTACACCTAACCCGGACCTAAACAGCTTCGTGATTAGGAGCAGTAACGCAGCTGACGTATCCCAGGTAGCCTGGGAGGTGTATCTCCtcgagcaccaccaccaccaccactgagatccggctgctaacaaagcccgaaaggaagctgagttggctgctgccaccgctgagcaataactagcataaccccttggggcctctaaacgggtcttgaggggttttttgctgaaaggaggaactatatccggattggcgaatgggacgcgccctgtagcggcgcattaagcgcggcgggtgtggtggttacgcgcagcgtgaccgctacacttgccagcgccctagcgcccgctcctttcgctttcttcccttcctttctcgccacgttcgccggctttccccgtcaagctctaaatcgggggctccctttagggttccgatttagtgctttacggcacctcgaccccaaaaaacttgattagggtgatggttcacgtagtgggccatcgccctgatagacggtttttcgccctttgacgttggagtccacgttctttaatagtggactcttgttccaaactggaacaacactcaaccctatctcggtctattcttttgatttataagggattttgccgatttcggcctattggttaaaaaatgagctgatttaacaaaaatttaacgcgaattttaacaaaatattaacgtttacaatttcaggtggcacttttcggggaaatgtgcgcggaacccctatttgtttatttttctaaatacattcaaatatgtatccgctcatgaattaattcttagaaaaactcatcgagcatcaaatgaaactgcaatttattcatatcaggattatcaataccatatttttgaaaaagccgtttctgtaatgaaggagaaaactcaccgaggcagttccataggatggcaagatcctggtatcggtctgcgattccgactcgtccaacatcaatacaacctattaatttcccctcgtcaaaaataaggttatcaagtgagaaatcaccatgagtgacgactgaatccggtgagaatggcaaaagtttatgcatttctttccagacttgttcaacaggccagccattacgctcgtcatcaaaatcactcgcatcaaccaaaccgttattcattcgtgattgcgcctgagcgagacgaaatacgcgatcgctgttaaaaggacaattacaaacaggaatcgaatgcaaccggcgcaggaacactgccagcgcatcaacaatattttcacctgaatcaggatattcttctaatacctggaatgctgttttcccggggatcgcagtggtgagtaaccatgcatcatcaggagtacggataaaatgcttgatggtcggaagaggcataaattccgtcagccagtttagtctgaccatctcatctgtaacatcattggcaacgctacctttgccatgtttcagaaacaactctggcgcatcgggcttcccatacaatcgatagattgtcgcacctgattgcccgacattatcgcgagcccatttatacccatataaatcagcatccatgttggaatttaatcgcggcctagagcaagacgtttcccgttgaatatggctcataacaccccttgtattactgtttatgtaagcagacagttttattgttcatgaccaaaatcccttaacgtgagttttcgttccactgagcgtcagaccccgtagaaaagatcaaaggatcttcttgagatcctttttttctgcgcgtaatctgctgcttgcaaacaaaaaaaccaccgctaccagcggtggtttgtttgccggatcaagagctaccaactctttttccgaaggtaactggcttcagcagagcgcagataccaaatactgtccttctagtgtagccgtagttaggccaccacttcaagaactctgtagcaccgcctacatacctcgctctgctaatcctgttaccagtggctgctgccagtggcgataagtcgtgtcttaccgggttggactcaagacgatagttaccggataaggcgcagcggtcgggctgaacggggggttcgtgcacacagcccagcttggagcgaacgacctacaccgaactgagatacctacagcgtgagctatgagaaagcgccacgcttcccgaagggagaaaggcggacaggtatccggtaagcggcagggtcggaacaggagagcgcacgagggagcttccagggggaaacgcctggtatctttatagtcctgtcgggtttcgccacctctgacttgagcgtcgatttttgtgatgctcgtcaggggggcggagcctatggaaaaacgccagcaacgcggcctttttacggttcctggccttttgctggccttttgctcacatgttctttcctgcgttatcccctgattctgtggataaccgtattaccgcctttgagtgagctgataccgctcgccgcagccgaacgaccgagcgcagcgagtcagtgagcgaggaagcggaagagcgcctgatgcggtattttctccttacgcatctgtgcggtatttcacaccgcatatatggtgcactctcagtacaatctgctctgatgccgcatagttaagccagtatacactccgctatcgctacgtgactgggtcatggctgcgccccgacacccgccaacacccgctgacgcgccctgacgggcttgtctgctcccggcatccgcttacagacaagctgtgaccgtctccgggagctgcatgtgtcagaggttttcaccgtcatcaccgaaacgcgcgaggcagctgcggtaaagctcatcagcgtggtcgtgaagcgattcacagatgtctgcctgttcatccgcgtccagctcgttgagtttctccagaagcgttaatgtctggcttctgataaagcgggccatgttaagggcggttttttcctgtttggtcactgatgcctccgtgtaagggggatttctgttcatgggggtaatgataccgatgaaacgagagaggatgctcacgatacgggttactgatgatgaacatgcccggttactggaacgttgtgagggtaaacaactggcggtatggatgcggcgggaccagagaaaaatcactcagggtcaatgccagcgcttcgttaatacagatgtaggtgttccacagggtagccagcagcatcctgcgatgcagatccggaacataatggtgcagggcgctgacttccgcgtttccagactttacgaaacacggaaaccgaagaccattcatgttgttgctcaggtcgcagacgttttgcagcagcagtcgcttcacgttcgctcgcgtatcggtgattcattctgctaaccagtaaggcaaccccgccagcctagccgggtcctcaacgacaggagcacgatcatgcgcacccgtggggccgccatgccggcgataatggcctgcttctcgccgaaacgtttggtggcgggaccagtgacgaaggcttgagcgagggcgtgcaagattccgaataccgcaagcgacaggccgatcatcgtcgcgctccagcgaaagcggtcctcgccgaaaatgacccagagcgctgccggcacctgtcctacgagttgcatgataaagaagacagtcataagtgcggcgacgatagtcatgccccgcgcccaccggaaggagctgactgggttgaaggctctcaagggcatcggtcgagatcccggtgcctaatgagtgagctaacttacattaattgcgttgcgctcactgcccgctttccagtcgggaaacctgtcgtgccagctgcattaatgaatcggccaacgcgcggggagaggcggtttgcgtattgggcgccagggtggtttttcttttcaccagtgagacgggcaacagctgattgcccttcaccgcctggccctgagagagttgcagcaagcggtccacgctggtttgccccagcaggcgaaaatcctgtttgatggtggttaacggcgggatataacatgagctgtcttcggtatcgtcgtatcccactaccgagatatccgcaccaacgcgcagcccggactcggtaatggcgcgcattgcgcccagcgccatctgatcgttggcaaccagcatcgcagtgggaacgatgccctcattcagcatttgcatggtttgttgaaaaccggacatggcactccagtcgccttcccgttccgctatcggctgaatttgattgcgagtgagatatttatgccagccagccagacgcagacgcgccgagacagaacttaatgggcccgctaacagcgcgatttgctggtgacccaatgcgaccagatgctccacgcccagtcgcgtaccgtcttcatgggagaaaataatactgttgatgggtgtctggtcagagacatcaagaaataacgccggaacattagtgcaggcagcttccacagcaatggcatcctggtcatccagcggatagttaatgatcagcccactgacgcgttgcgcgagaagattgtgcaccgccgctttacaggcttcgacgccgcttcgttctaccatcgacaccaccacgctggcacccagttgatcggcgcgagatttaatcgccgcgacaatttgcgacggcgcgtgcagggccagactggaggtggcaacgccaatcagcaacgactgtttgcccgccagttgttgtgccacgcggttgggaatgtaattcagctccgccatcgccgcttccactttttcccgcgttttcgcagaaacgtggctggcctggttcaccacgcgggaaacggtctgataagagacaccggcatactctgcgacatcgtataacgttactggtttcacattcaccaccctgaattgactctcttccgggcgctatcatgccataccgcgaaaggttttgcgccattcgatggtgtccgggatctcgacgctctcccttatgcgactcctgcattaggaagcagcccagtagtaggttgaggccgttgagcaccgccgccgcaaggaatggtgcatgcaaggagatggcgcccaacagtcccccggccacggggcctgccaccatacccacgccgaaacaagcgctcatgagcccgaagtggcgagcccgatcttccccatcggtgatgtcggcg

GBH038_054_pET26b+:

atataggcgccagcaaccgcacctgtggcgccggtgatgccggccacgatgcgtccggcgtagaggatcgagatctcgatcccgcgaaattaatacgactcactataggggaattgtgagcggataacaattcccctctagaaataattttgtttaactttaagaaggagatatacatatgaaatacctgctgccgaccgctgctgctggtctgctgctcctcgctgcccagccggcgatggccatggatatcggaattaattcggatccgaattcgagctccgtcgacaagcttATGGCACTAGTAGATTTAGTGAGGGCTGGGGGATATTCTATTGAGTACCCGCAATTCTCCAGTATGGCTAAGCTAAAAGAGTTCCCACACTCTGAGGACGGGAAACTTGTTAGGTTGTTGTCTTGGCATGAAGGGGTTGGTTTAGGTGGTGGGCTGTTTAAGGTCAGCACTAGCAGCACCGCTACAGGTAACGACGGTACTGTAGTAGTAGCTAGTAATGGGGTGCGGCTGCTTCGAGTAGTAAACGGACCTATCTGGGCGGATATGTTTGGTGCACTACCGAATTCAGACATAGACAGTATGCCAGCAGTAGCTGCGGCTTATGCGTACGCTGCTTCTGTGAATACGGACCTGTATATAGGGGTGGCGACTTACAAATTCAAGGGGAGCACCCCAATTAATATAGACCCATCCAGGGCCGGTATTATTGGTTATCAAGGTAAGGTGCGCATTGACTGCTCCGAGTTTACTGGCTCGATTGTGTTTTCTATAAACAGCAGCTATAGCTACACCCCCGCAGCCTACTACAACAACCTTAGTCCGGCCCTGCAGGGGCTGTACGTGTTGGGTGCTAAAACGTCCGGTGTAGACGGTCTACTGGTTGGTAGGGAAACAGTGGGGGCGGACAAGAGCTATAACGGGCAGACAGAGATTCGTGAGTGCACGTTCGATAAGTTCGACCGCAACATCCGAATGGGGCACAACTCTTGGCGCTTTGTGTTCTACAAAGTAAACAGCCTAAACGCTCTCAGTCCTAACGGGATACTGTACGTACCGGCTGGGCTGGATGACTCTGGGGAGATTCTGTCGTTCTACCACTGCCAGTTCTTTGACGGTGCAGGTAGTAATATCCGATTATCTTGCTCCTCGTATACTATGGTATTTAATACTTGCTCGTTCCTGAACATCACGTTCTTTGTGGATTCGGCGAGTAGTGCGACAGTAACTTGCAATGGGTGCAACTTCGAGAACCCGGGAAGTGCTAGTACCCGTAGATACGTTGACATTAGTGCTGGGCACACTAACGTATTCAACATTATTGGGGGCAGTATAGTTACAAACAGCAACCCTGGACAGACTCAGGCGCTGCTGTATGTCTCTACCGACAACCTACTGAACCTTGTAGGTGTAACTGTTCCTTACGGCGGGCACTACCAGCAGGAGCAGGAGCTCGGGTATCACGCATTCATCGGAGGGGCCGGTACAGTCACAACCTCTGGAGTTATGCTGCAGCTACGCAACGGGGCGGGTACGTGTCCTTTGCACTCTAGTCTTAGCACATTCAGTAACTGGGATTTTGGTTATGGGAACCTGAATGCTTGGACGGTAGATAAGGGGACTGGTACATCCTCTGTGGTAGAGTATCTGGCTAATGCTGGACCTAAAGGTACAGAGGGAGCTATGCGAGTTGCTCCTGTAAGTGTCGGTACCAACATATCGCAAGTACAGGCAGTAACTAACCCTGGCATGTTCAGTATGTCCTGCATGGTGAACATTGCGACGACCTCCGGTAATGCAGGGCAGATATCTATTGGGTTCTTGGATGCTGCAGGTAACAGCCTGCCTGGTGGGGTAGCAGCTAACCTGGGTACCACCACGGGTTGGAAGGTAATCGGCAAGAACACCTTGAGGGGCAAAGTCCCAATCGGTGCAAAGCAGATTCGTGTGAACATTCAGACCGTGGCCGGTGCAGACGTTAAGTACGCGTACTTGCTGTGCAACGTGGTCAAGCtcgagcaccaccaccaccaccactgagatccggctgctaacaaagcccgaaaggaagctgagttggctgctgccaccgctgagcaataactagcataaccccttggggcctctaaacgggtcttgaggggttttttgctgaaaggaggaactatatccggattggcgaatgggacgcgccctgtagcggcgcattaagcgcggcgggtgtggtggttacgcgcagcgtgaccgctacacttgccagcgccctagcgcccgctcctttcgctttcttcccttcctttctcgccacgttcgccggctttccccgtcaagctctaaatcgggggctccctttagggttccgatttagtgctttacggcacctcgaccccaaaaaacttgattagggtgatggttcacgtagtgggccatcgccctgatagacggtttttcgccctttgacgttggagtccacgttctttaatagtggactcttgttccaaactggaacaacactcaaccctatctcggtctattcttttgatttataagggattttgccgatttcggcctattggttaaaaaatgagctgatttaacaaaaatttaacgcgaattttaacaaaatattaacgtttacaatttcaggtggcacttttcggggaaatgtgcgcggaacccctatttgtttatttttctaaatacattcaaatatgtatccgctcatgaattaattcttagaaaaactcatcgagcatcaaatgaaactgcaatttattcatatcaggattatcaataccatatttttgaaaaagccgtttctgtaatgaaggagaaaactcaccgaggcagttccataggatggcaagatcctggtatcggtctgcgattccgactcgtccaacatcaatacaacctattaatttcccctcgtcaaaaataaggttatcaagtgagaaatcaccatgagtgacgactgaatccggtgagaatggcaaaagtttatgcatttctttccagacttgttcaacaggccagccattacgctcgtcatcaaaatcactcgcatcaaccaaaccgttattcattcgtgattgcgcctgagcgagacgaaatacgcgatcgctgttaaaaggacaattacaaacaggaatcgaatgcaaccggcgcaggaacactgccagcgcatcaacaatattttcacctgaatcaggatattcttctaatacctggaatgctgttttcccggggatcgcagtggtgagtaaccatgcatcatcaggagtacggataaaatgcttgatggtcggaagaggcataaattccgtcagccagtttagtctgaccatctcatctgtaacatcattggcaacgctacctttgccatgtttcagaaacaactctggcgcatcgggcttcccatacaatcgatagattgtcgcacctgattgcccgacattatcgcgagcccatttatacccatataaatcagcatccatgttggaatttaatcgcggcctagagcaagacgtttcccgttgaatatggctcataacaccccttgtattactgtttatgtaagcagacagttttattgttcatgaccaaaatcccttaacgtgagttttcgttccactgagcgtcagaccccgtagaaaagatcaaaggatcttcttgagatcctttttttctgcgcgtaatctgctgcttgcaaacaaaaaaaccaccgctaccagcggtggtttgtttgccggatcaagagctaccaactctttttccgaaggtaactggcttcagcagagcgcagataccaaatactgtccttctagtgtagccgtagttaggccaccacttcaagaactctgtagcaccgcctacatacctcgctctgctaatcctgttaccagtggctgctgccagtggcgataagtcgtgtcttaccgggttggactcaagacgatagttaccggataaggcgcagcggtcgggctgaacggggggttcgtgcacacagcccagcttggagcgaacgacctacaccgaactgagatacctacagcgtgagctatgagaaagcgccacgcttcccgaagggagaaaggcggacaggtatccggtaagcggcagggtcggaacaggagagcgcacgagggagcttccagggggaaacgcctggtatctttatagtcctgtcgggtttcgccacctctgacttgagcgtcgatttttgtgatgctcgtcaggggggcggagcctatggaaaaacgccagcaacgcggcctttttacggttcctggccttttgctggccttttgctcacatgttctttcctgcgttatcccctgattctgtggataaccgtattaccgcctttgagtgagctgataccgctcgccgcagccgaacgaccgagcgcagcgagtcagtgagcgaggaagcggaagagcgcctgatgcggtattttctccttacgcatctgtgcggtatttcacaccgcatatatggtgcactctcagtacaatctgctctgatgccgcatagttaagccagtatacactccgctatcgctacgtgactgggtcatggctgcgccccgacacccgccaacacccgctgacgcgccctgacgggcttgtctgctcccggcatccgcttacagacaagctgtgaccgtctccgggagctgcatgtgtcagaggttttcaccgtcatcaccgaaacgcgcgaggcagctgcggtaaagctcatcagcgtggtcgtgaagcgattcacagatgtctgcctgttcatccgcgtccagctcgttgagtttctccagaagcgttaatgtctggcttctgataaagcgggccatgttaagggcggttttttcctgtttggtcactgatgcctccgtgtaagggggatttctgttcatgggggtaatgataccgatgaaacgagagaggatgctcacgatacgggttactgatgatgaacatgcccggttactggaacgttgtgagggtaaacaactggcggtatggatgcggcgggaccagagaaaaatcactcagggtcaatgccagcgcttcgttaatacagatgtaggtgttccacagggtagccagcagcatcctgcgatgcagatccggaacataatggtgcagggcgctgacttccgcgtttccagactttacgaaacacggaaaccgaagaccattcatgttgttgctcaggtcgcagacgttttgcagcagcagtcgcttcacgttcgctcgcgtatcggtgattcattctgctaaccagtaaggcaaccccgccagcctagccgggtcctcaacgacaggagcacgatcatgcgcacccgtggggccgccatgccggcgataatggcctgcttctcgccgaaacgtttggtggcgggaccagtgacgaaggcttgagcgagggcgtgcaagattccgaataccgcaagcgacaggccgatcatcgtcgcgctccagcgaaagcggtcctcgccgaaaatgacccagagcgctgccggcacctgtcctacgagttgcatgataaagaagacagtcataagtgcggcgacgatagtcatgccccgcgcccaccggaaggagctgactgggttgaaggctctcaagggcatcggtcgagatcccggtgcctaatgagtgagctaacttacattaattgcgttgcgctcactgcccgctttccagtcgggaaacctgtcgtgccagctgcattaatgaatcggccaacgcgcggggagaggcggtttgcgtattgggcgccagggtggtttttcttttcaccagtgagacgggcaacagctgattgcccttcaccgcctggccctgagagagttgcagcaagcggtccacgctggtttgccccagcaggcgaaaatcctgtttgatggtggttaacggcgggatataacatgagctgtcttcggtatcgtcgtatcccactaccgagatatccgcaccaacgcgcagcccggactcggtaatggcgcgcattgcgcccagcgccatctgatcgttggcaaccagcatcgcagtgggaacgatgccctcattcagcatttgcatggtttgttgaaaaccggacatggcactccagtcgccttcccgttccgctatcggctgaatttgattgcgagtgagatatttatgccagccagccagacgcagacgcgccgagacagaacttaatgggcccgctaacagcgcgatttgctggtgacccaatgcgaccagatgctccacgcccagtcgcgtaccgtcttcatgggagaaaataatactgttgatgggtgtctggtcagagacatcaagaaataacgccggaacattagtgcaggcagcttccacagcaatggcatcctggtcatccagcggatagttaatgatcagcccactgacgcgttgcgcgagaagattgtgcaccgccgctttacaggcttcgacgccgcttcgttctaccatcgacaccaccacgctggcacccagttgatcggcgcgagatttaatcgccgcgacaatttgcgacggcgcgtgcagggccagactggaggtggcaacgccaatcagcaacgactgtttgcccgccagttgttgtgccacgcggttgggaatgtaattcagctccgccatcgccgcttccactttttcccgcgttttcgcagaaacgtggctggcctggttcaccacgcgggaaacggtctgataagagacaccggcatactctgcgacatcgtataacgttactggtttcacattcaccaccctgaattgactctcttccgggcgctatcatgccataccgcgaaaggttttgcgccattcgatggtgtccgggatctcgacgctctcccttatgcgactcctgcattaggaagcagcccagtagtaggttgaggccgttgagcaccgccgccgcaaggaatggtgcatgcaaggagatggcgcccaacagtcccccggccacggggcctgccaccatacccacgccgaaacaagcgctcatgagcccgaagtggcgagcccgatcttccccatcggtgatgtcggcg

GBH019_279_pET26b+:

atataggcgccagcaaccgcacctgtggcgccggtgatgccggccacgatgcgtccggcgtagaggatcgagatctcgatcccgcgaaattaatacgactcactataggggaattgtgagcggataacaattcccctctagaaataattttgtttaactttaagaaggagatatacatatgaaatacctgctgccgaccgctgctgctggtctgctgctcctcgctgcccagccggcgatggccatggatatcggaattaattcggatccgaattcgagctccgtcgacaagcttATGGCGAATGAATTAATCCAACCCAAAGGTTCAGTATCAAGAGAGACGAATAAAGAATCTATAGCTAGAATATTTGGTATTAAAAAATCTTCGGTAGGATATCTATCTACTTCAGTAGAAGTAGACTTATATACAATTTTATATGATGAAAACACACAAACTTGTTGGTATAGGAGCACTGCAACAGGAATACCTGTATCGTGGAGTATCTCAGGACAAAATTTAACTTTAATTACTGCTGTAGGTACATTTGGTTTAGAAAAAGCCCCCTCTATTTTAGATTTATCTACAAATATTGGTGCAAGTAAAATAGGGTTATCTCAAGGTGGAACAGTTCAAAGCGTATTAACAGAGATACCTGCCTCAAAATTTGGTGTAACAGGTGACGGTGTTGCCTGTGGTTCTGCATGTGTAGCGGCTATTGAATATATTATGCAGAATGGTGGAACACTAATATTTCCTAAAGGTGAGGTGAACTGGGGAACTACTCGCCATCAATTTGCTATCTATAATGGCAAGCCTTGTACTATCAAAGGTTCTGATGGTGGAACCACATGGACATTCGATAACGTTGATCCGGTTGCTAATGCCACAGGTGCAGCATGGCCGTTCAGTGAACCATTCCTTGTTGAATTTGGTGGACAGCCAACCGCACAAGGCGTCTTTGTGGAGCCAGTAACTGTTGAAAATCTCAGTATCGACTATACCCGTCAAGCTAATAAAGGTGGGCCTACATATGAGACGATGGGGGTGGGAGCACATCCTACACCATATTCGGATGGCACTCTTGGTCTGCGTTTTAGCTATTGTCGATCACCCGTCGTGCGTAACGTCACGATGAGAGAGATCTACGGGTCAGGTATTCAGTTTTGGAAATGCTCGATGGCTCTGGCAGAAAACAACAGTCTATACAACGTATCTGCCAACCAACCTTTAGGGTCAAGTGGTAGTGAGTCTGTCGATCACTTTGGTTGGGCTATCTGGTCTGGTGCCAGCCCAAAAACGATTATCCGACGTAACACCGCGATTAATAAGCGTGTTTTCGTATGCGATCCAGCATTGAAATCCCCTAACAACAACGTGGTATATAATGGTACTCTTTGTGGATATATCGGCATTTTCGCGGAGTACGGGAGCAATGGTGGTACTTCCACAATCTATCCACCTGACTTTGAATGGCAGTCGGATTCTTCCGCCGATAAGAGAACCTACATTACACTGGAAGATAACCTCGTATGGGGTTACACTATGTCCATTAAGTCTGAGGCAACCACCTCGGCTCGAATCATTAATAATACGCTGCTGAACCACTATATCGGTGTATCTGTTCAGGCATCAGCCGATGTTGTAGGTAACTACATCAATGGATTACAGGCTGATTTGCAAAAATGCCCACAGAACGGATTTGAATTGCAGCGTGGTGGGATTATGCTTTCCTGGTGGGCATCATCTGTGCAAGATCACCGACAGTATGTAGCTCAAAACTATGTTTATTCTGCTGCGTACAACTGCATTTCCATTGGTAAGGCTTACGCCACAATAGATGATAACCAGTTTACAATCACAGGCACAGCCAGATTTATAAACTCGGTTACTTCCTTCAACGTAGACTTGCTGGAGATGAGAGGGAACAAGCTCCTGATGTCTGAAAATTGCACACAGACTGTTCCTATGCGTGTCACTAACTGTTCTAAGGTTGTGATGGAAGCCAACTTTATAGAAAATAAAAGCACCACAGCCCGTATGTCAGTGCAGATGGTCAACCCTGTAATACGTAACAATACTTTCAAAGGGTGGGTTCAGATATACCTGTTATCAGCAGGCAGCATAGTGGAAGAGAATATATCCACTGATGATACTAGCAATAAAGGGTTAGTAATTCAGGTTGCATCAGCGGCTGACTGCATTATACGCAGGAACCAGATTACAAAGTATAATGCTGACGATGAGGATCAAATAATTTTCTTATCCAATGCTGCGAGAGCTATCATCGATAGTAATATCATAAACTATGCTGCAACTACTGGTTCGGGAAGAACTAAGAGTATCCCAGTAATCAAAACATATGGCACGTGCTTTTACACCAGAATACTCAACAACAGGGTTATTGGTGATAGTAGTGCTACATTCGGTTTTACGCTTTTCGATGGTTCATCGGGGGCGAGAGTACTGGAATGTCGTGGTAACGGGACAGATAGTACAACCAGACAGATGTTCACAATGTATTCTCAAAGTGGTCCATGGTTTGTTTCTGGAAACGACTGGACACAGACATATAACTCTGAGATAAATACCGCAGCTAATATATATGCATCATATAAACCAGCCCAAGGCGAGAAAGTACCTTATCTACGAGCACAGGCAGGGGGTGCTGAAGGTATAGTGTATGTGGGAACTGGCTGGCTAACTTATGGTTCCATATCTTCTCtcgagcaccaccaccaccaccactgagatccggctgctaacaaagcccgaaaggaagctgagttggctgctgccaccgctgagcaataactagcataaccccttggggcctctaaacgggtcttgaggggttttttgctgaaaggaggaactatatccggattggcgaatgggacgcgccctgtagcggcgcattaagcgcggcgggtgtggtggttacgcgcagcgtgaccgctacacttgccagcgccctagcgcccgctcctttcgctttcttcccttcctttctcgccacgttcgccggctttccccgtcaagctctaaatcgggggctccctttagggttccgatttagtgctttacggcacctcgaccccaaaaaacttgattagggtgatggttcacgtagtgggccatcgccctgatagacggtttttcgccctttgacgttggagtccacgttctttaatagtggactcttgttccaaactggaacaacactcaaccctatctcggtctattcttttgatttataagggattttgccgatttcggcctattggttaaaaaatgagctgatttaacaaaaatttaacgcgaattttaacaaaatattaacgtttacaatttcaggtggcacttttcggggaaatgtgcgcggaacccctatttgtttatttttctaaatacattcaaatatgtatccgctcatgaattaattcttagaaaaactcatcgagcatcaaatgaaactgcaatttattcatatcaggattatcaataccatatttttgaaaaagccgtttctgtaatgaaggagaaaactcaccgaggcagttccataggatggcaagatcctggtatcggtctgcgattccgactcgtccaacatcaatacaacctattaatttcccctcgtcaaaaataaggttatcaagtgagaaatcaccatgagtgacgactgaatccggtgagaatggcaaaagtttatgcatttctttccagacttgttcaacaggccagccattacgctcgtcatcaaaatcactcgcatcaaccaaaccgttattcattcgtgattgcgcctgagcgagacgaaatacgcgatcgctgttaaaaggacaattacaaacaggaatcgaatgcaaccggcgcaggaacactgccagcgcatcaacaatattttcacctgaatcaggatattcttctaatacctggaatgctgttttcccggggatcgcagtggtgagtaaccatgcatcatcaggagtacggataaaatgcttgatggtcggaagaggcataaattccgtcagccagtttagtctgaccatctcatctgtaacatcattggcaacgctacctttgccatgtttcagaaacaactctggcgcatcgggcttcccatacaatcgatagattgtcgcacctgattgcccgacattatcgcgagcccatttatacccatataaatcagcatccatgttggaatttaatcgcggcctagagcaagacgtttcccgttgaatatggctcataacaccccttgtattactgtttatgtaagcagacagttttattgttcatgaccaaaatcccttaacgtgagttttcgttccactgagcgtcagaccccgtagaaaagatcaaaggatcttcttgagatcctttttttctgcgcgtaatctgctgcttgcaaacaaaaaaaccaccgctaccagcggtggtttgtttgccggatcaagagctaccaactctttttccgaaggtaactggcttcagcagagcgcagataccaaatactgtccttctagtgtagccgtagttaggccaccacttcaagaactctgtagcaccgcctacatacctcgctctgctaatcctgttaccagtggctgctgccagtggcgataagtcgtgtcttaccgggttggactcaagacgatagttaccggataaggcgcagcggtcgggctgaacggggggttcgtgcacacagcccagcttggagcgaacgacctacaccgaactgagatacctacagcgtgagctatgagaaagcgccacgcttcccgaagggagaaaggcggacaggtatccggtaagcggcagggtcggaacaggagagcgcacgagggagcttccagggggaaacgcctggtatctttatagtcctgtcgggtttcgccacctctgacttgagcgtcgatttttgtgatgctcgtcaggggggcggagcctatggaaaaacgccagcaacgcggcctttttacggttcctggccttttgctggccttttgctcacatgttctttcctgcgttatcccctgattctgtggataaccgtattaccgcctttgagtgagctgataccgctcgccgcagccgaacgaccgagcgcagcgagtcagtgagcgaggaagcggaagagcgcctgatgcggtattttctccttacgcatctgtgcggtatttcacaccgcatatatggtgcactctcagtacaatctgctctgatgccgcatagttaagccagtatacactccgctatcgctacgtgactgggtcatggctgcgccccgacacccgccaacacccgctgacgcgccctgacgggcttgtctgctcccggcatccgcttacagacaagctgtgaccgtctccgggagctgcatgtgtcagaggttttcaccgtcatcaccgaaacgcgcgaggcagctgcggtaaagctcatcagcgtggtcgtgaagcgattcacagatgtctgcctgttcatccgcgtccagctcgttgagtttctccagaagcgttaatgtctggcttctgataaagcgggccatgttaagggcggttttttcctgtttggtcactgatgcctccgtgtaagggggatttctgttcatgggggtaatgataccgatgaaacgagagaggatgctcacgatacgggttactgatgatgaacatgcccggttactggaacgttgtgagggtaaacaactggcggtatggatgcggcgggaccagagaaaaatcactcagggtcaatgccagcgcttcgttaatacagatgtaggtgttccacagggtagccagcagcatcctgcgatgcagatccggaacataatggtgcagggcgctgacttccgcgtttccagactttacgaaacacggaaaccgaagaccattcatgttgttgctcaggtcgcagacgttttgcagcagcagtcgcttcacgttcgctcgcgtatcggtgattcattctgctaaccagtaaggcaaccccgccagcctagccgggtcctcaacgacaggagcacgatcatgcgcacccgtggggccgccatgccggcgataatggcctgcttctcgccgaaacgtttggtggcgggaccagtgacgaaggcttgagcgagggcgtgcaagattccgaataccgcaagcgacaggccgatcatcgtcgcgctccagcgaaagcggtcctcgccgaaaatgacccagagcgctgccggcacctgtcctacgagttgcatgataaagaagacagtcataagtgcggcgacgatagtcatgccccgcgcccaccggaaggagctgactgggttgaaggctctcaagggcatcggtcgagatcccggtgcctaatgagtgagctaacttacattaattgcgttgcgctcactgcccgctttccagtcgggaaacctgtcgtgccagctgcattaatgaatcggccaacgcgcggggagaggcggtttgcgtattgggcgccagggtggtttttcttttcaccagtgagacgggcaacagctgattgcccttcaccgcctggccctgagagagttgcagcaagcggtccacgctggtttgccccagcaggcgaaaatcctgtttgatggtggttaacggcgggatataacatgagctgtcttcggtatcgtcgtatcccactaccgagatatccgcaccaacgcgcagcccggactcggtaatggcgcgcattgcgcccagcgccatctgatcgttggcaaccagcatcgcagtgggaacgatgccctcattcagcatttgcatggtttgttgaaaaccggacatggcactccagtcgccttcccgttccgctatcggctgaatttgattgcgagtgagatatttatgccagccagccagacgcagacgcgccgagacagaacttaatgggcccgctaacagcgcgatttgctggtgacccaatgcgaccagatgctccacgcccagtcgcgtaccgtcttcatgggagaaaataatactgttgatgggtgtctggtcagagacatcaagaaataacgccggaacattagtgcaggcagcttccacagcaatggcatcctggtcatccagcggatagttaatgatcagcccactgacgcgttgcgcgagaagattgtgcaccgccgctttacaggcttcgacgccgcttcgttctaccatcgacaccaccacgctggcacccagttgatcggcgcgagatttaatcgccgcgacaatttgcgacggcgcgtgcagggccagactggaggtggcaacgccaatcagcaacgactgtttgcccgccagttgttgtgccacgcggttgggaatgtaattcagctccgccatcgccgcttccactttttcccgcgttttcgcagaaacgtggctggcctggttcaccacgcgggaaacggtctgataagagacaccggcatactctgcgacatcgtataacgttactggtttcacattcaccaccctgaattgactctcttccgggcgctatcatgccataccgcgaaaggttttgcgccattcgatggtgtccgggatctcgacgctctcccttatgcgactcctgcattaggaagcagcccagtagtaggttgaggccgttgagcaccgccgccgcaaggaatggtgcatgcaaggagatggcgcccaacagtcccccggccacggggcctgccaccatacccacgccgaaacaagcgctcatgagcccgaagtggcgagcccgatcttccccatcggtgatgtcggcg
